# Supplementary material for: A versatile and customizable low-cost 3D-printed open standard for microscopic imaging
Source: Nat Commun. 2020 Nov 25;11:5979. doi: 10.1038/s41467-020-19447-9 (PMC7688980; doi:10.1038/s41467-020-19447-9)
Supplement: Supplementary file 1 — Supplementary Information [file 41467_2020_19447_MOESM1_ESM.pdf]

Supplementary Information

A Versatile and Customizable Low-Cost 3D-Printed Open Standard for Microscopic Imaging

Benedict Diederich, René Lachmann, Swen Carlstedt, Barbora Marsikova, Haoran Wang, Xavier Uwurukundo, Alexander Mosig, Rainer Heintzmann

Contents

1 Supplementary Figure 1. . . . 3
2 Supplementary Figure 2. . . . 4
3 Supplementary Figure 3. . . . 5
1 Supplementary Video 1. . . . 6
2 Supplementary Video 2. . . . 6
3 Supplementary Video 3. . . . 6
4 Supplementary Video 4. . . . 6
5 Supplementary Video 5. . . . 7
6 Supplementary Video 6. . . . 7
7 Supplementary Video 7. . . . 7
1 Module Developer Kit 8
2 The Cube 11
3 The Baseplate 11
4 Cube Inserts 12
5 Good Practice to Transfer an Optical System to UC2 13
6 Software 15
6.1 Details about Hardware and Software Control 15
7 Experimental Details 17
7.1 Long-Term In-Incubator Microscopy 17
7.2 Optical Resolution, System and Long Term Stability 19
7.3 Autofocus for long-term in-focus measurements 21
7.4 Optical resolution of fluorescent data using FRC 22
7.5 Compare read noise between Raspberry Pi and Huawei P20 Pro camera . 24
7.6 Quantitative Phase: aIDT 27
7.7 Light Sheet Microscope 27
7.8 Conversion from UC2 Incubator to Lightsheet Microscope 30
7.9 Image Scanning Microscope (*open*ISM) 31
7.10 Quantitative Imaging using *open*KOEHLER 33
7.11 Educational Areas 34
8 Bill of material 37
9 UC2 Use-cases 39
10 Sample Preparation 40
10.1 Primary Macrophages 40
10.2 Phagocytosis: 41
10.3 HPMEC 41
10.4 Zebrafish larva for light sheet imaging 41
10.5 Drosophila larva for light sheet imaging 41
10.6 *E. coli* bacteria 42

Supplementary Figure

1 Supplementary Figure 1.

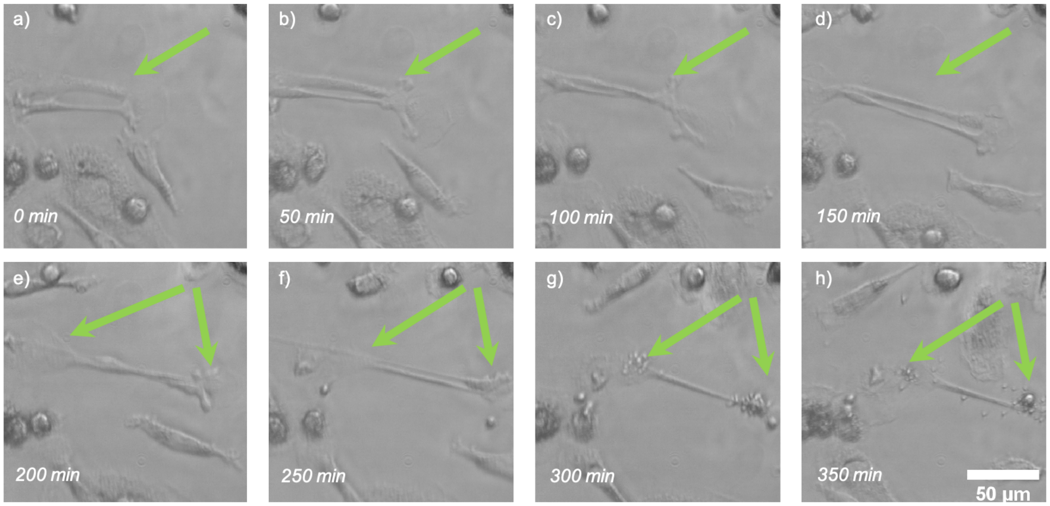

Figure 1. Cell-Apoptosis of macrophages From image series acquired using the incubator microscope (10 ×, 0.32 NA objective) at a frame-rate of 1 frame/minute. a)-d)One observed cell first became elongated and e)-f) started blebbing, a clear sign of apoptosis. g)-h) The fragmentation of the cell to apoptotic bodies is clearly visible. The cell fragments are then cleared via efferocytosis by other macrophages (see Supp. Video 6 from 3:10, left). The displayed images are temporally spaced by 50min each.

2 Supplementary Figure 2.

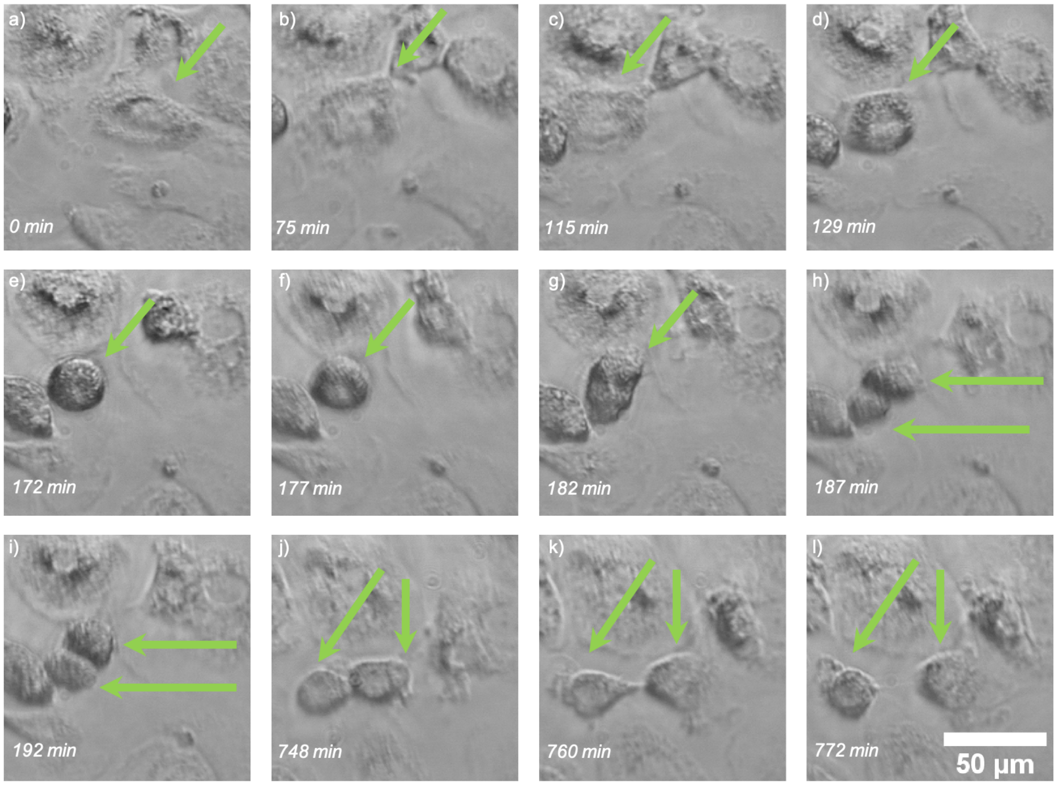

Figure 2. Cell Division of Macrophages - Long-term (48 h) image series acquired using the incubator microscope (10 ×, 0.32 NA objective) at a frame-rate of 1 frame/minute. A very rare cell division of a macrophage was observed. a)-d) The cell stopped, e)-f) constricted and g)-l) divided into two cells. Measurement time-points in minutes (min) from starting point a) = 5days 18hours 33min are: 75min, 115min, 129min, 172min, 177min, 182min, 187min, 192min, 748min, 760min, 774min. (see Supp. Video 6 from 7:04 - 7:08, centre right)

3 Supplementary Figure 3.

43

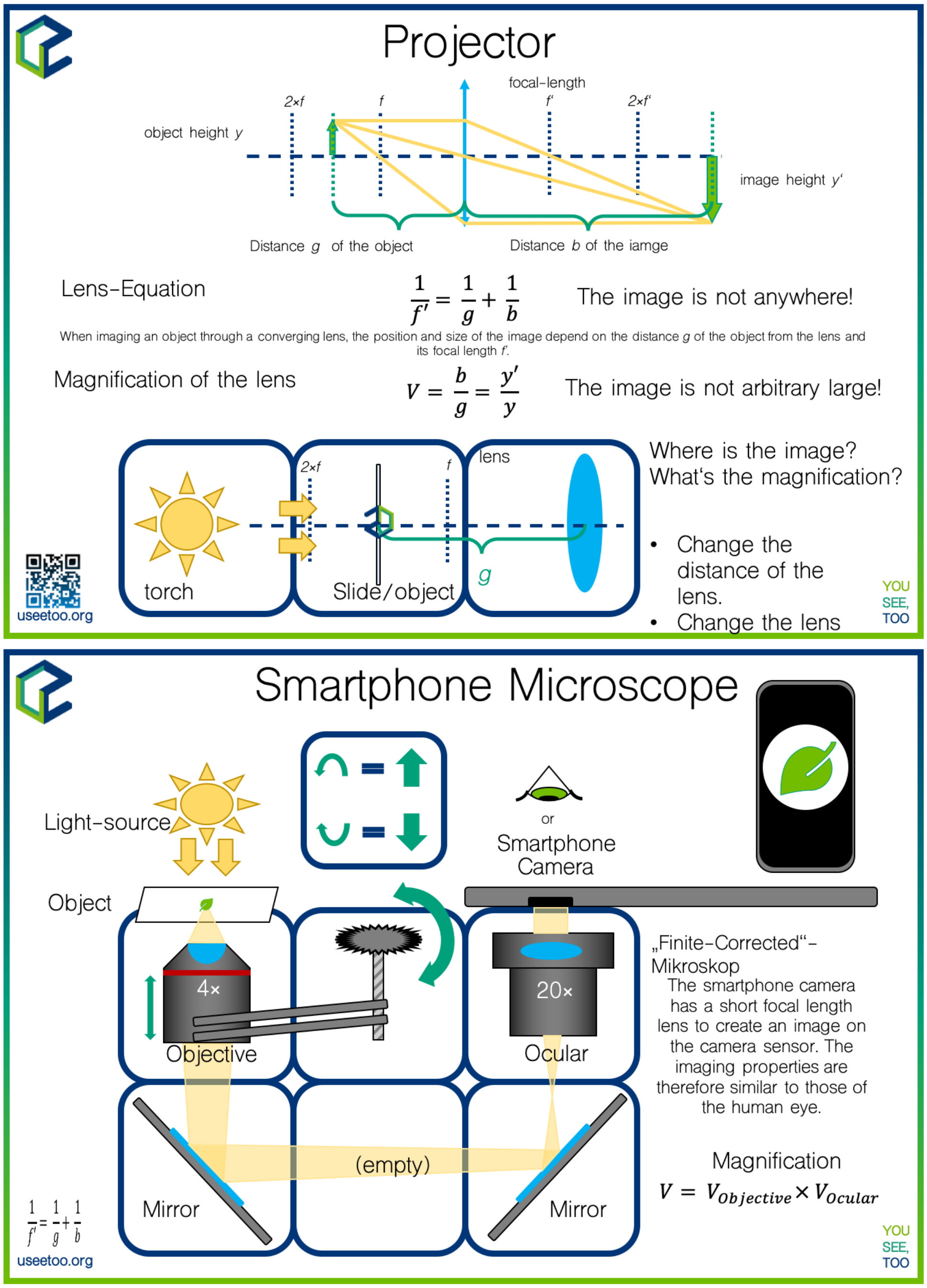

Figure 3. Educational chart Exemplary slides to show the basic properties of a simple projector or a smartphone-based microscope for the use in schools. The optical layout acts as a printed template for the different cubes. Students can conveniently place the cubes in these place-holders to create the microscope and observe an image using their eyes or cellphones and discuss the results.

Supplementary Video

1 Supplementary Video 1.

Long-term ($48\,h$) image series with the incubator microscope ($10\,\times$, 0.32 NA objective) at a frame-rate of 1 frame/minute. Incubator-contained measurement of isolated human blood monocytes. The aim was to document differentiation of monocytes to macrophages and analyse their movement pattern without stimulation.

2 Supplementary Video 2.

The video shows long-term imaging of the differentiation of blood-born monocytes to macrophages. Within the time span of seven days the monocytes increase size and are “looking” around. Obvious are the filopodia surround the cells. Moving macrophages become fusiform, elongate and follow their protrusions with the cell body.

3 Supplementary Video 3.

Reconstruction of the complex refractive index of unlabelled cheek cells using the annular intensity diffraction tomography algorithm (aIDT). A number of LEDs on an LED-ring placed at a distance to the sample of $\approx 74\,mm$ such that local illumination is approximated as a series of plane waves varying in azimuth. The inverse filtering process can reconstruct a 3D stack of the permittivity distribution. The acquisition was performed using a cellphone camera (Huawei P20 Pro, China) further described in Chapter 7.6.

4 Supplementary Video 4.

Through-focus series of a Drosophila larva. Due to the large depth of field of the $4\times$, NA=0.17 objective, a data stack was acquired by moving the light sheet through a fixed sample i.e. by changing the angle of the kinematic mirror in the illumination path. The GFP-expressing drosophila larva was focussed by the detection path and the illumination plane was then moved through it by changing the tilt of the kinematic mirror. Although the whole three-dimensional sample is in focus, only the illuminated parts yield signal being imaged onto the camera. The video was acquired with a cellphone camera (Huawei P20 Pro, China) and a $20\times$ eyepiece.

Alternatively, one can move the whole sample through the fixed light sheet aligned to the focus-plane using the sample-stage equipped with a flexure bearing. This was done in the video of the GFP-expressing zebrafish larva. The video was acquired with a Raspberry Pi camera with a lens and a $20\times$ eyepiece.

5 Supplementary Video 5.

The conversion from a simple bright field into a light sheet microscope can be accomplished within less than five minutes using TheBOX. The modules can easily be reused for different imaging modalities. The components are pre-aligned and remain their position when packed again, useful for transporting the whole system.

6 Supplementary Video 6.

Long-term measurements of MDCK-cells at room temperature over night $(8\,h)$ in an $35\,mm$ petri-dish at a UC2 workshop , which took place in Oslo, where participants were able to bring their samples.

7 Supplementary Video 7.

Time-series imaging at $\approx 1\,fps$ of fixed but mobile *E. coli* bacteria using the infinity-corrected fluorescence microscope (see Supp. Section 7.4). The ATTO647-labelled *E. coli* were illuminated with a coherent entertainment laser ($\lambda_{red} = 635/637\,nm$, $P_{laser} = 200\,mW$) move in aqueous solution due to Brownian motion and can nicely be observed with the low-SNR RGB camera from the Raspberry Pi (v2.1). During the ca. 10 minutes experiment, some bacteria start adhering to the cover glass. By increasing the laser intensity inside the UC2 GUI, a dominant bleaching of the bacteria can be observed.

Supplementary Notes

1 Module Developer Kit

One aspect which is missing in many open-source and open-science projects is the ability to interact with the project in order to introduce own modifications to individual needs. During our study we found that one major requirement in order to provide users easy access to the resources and to make it attractive to start developing on an open project - like the proposed UC2 system - is an easy to understand documentation. It should provide an intuitive way into the project to reduce the inhibition threshold to be engaged.
Inspired by the recently discontinued modular cellphone project *ARA* by Google Inc. [1] we created a comprehensive document called the Module Developer Kit (MDK, GitHub repository) which describes the good practice of cube-design and customized inserts. This includes the CAD-files for common CAD software like Autodesk Inventor 2019 (Autodesk ®Inventor LT™) OpenSCAD (`www.openscad.org`, v2019.05), as well as schematics to port the design to other software tools. It emphasizes the idea of having the UC2-system as a supporting base-structure or skeleton to become a common standard for a large variety of different components of different manufactures. Having a "zoo" or library of modules developed by an active community which are useful for many people guarantees a long lifetime of the project. All files can be found in our hard- and software repository [2, 3].

At first we introduce the naming-convention of the UC2 system to give a better understanding of the module hierarchy. These terms are defined in the table (1) below and illustrated in Fig. 4. Based on these modules and inserts, a complex optical system can be created.

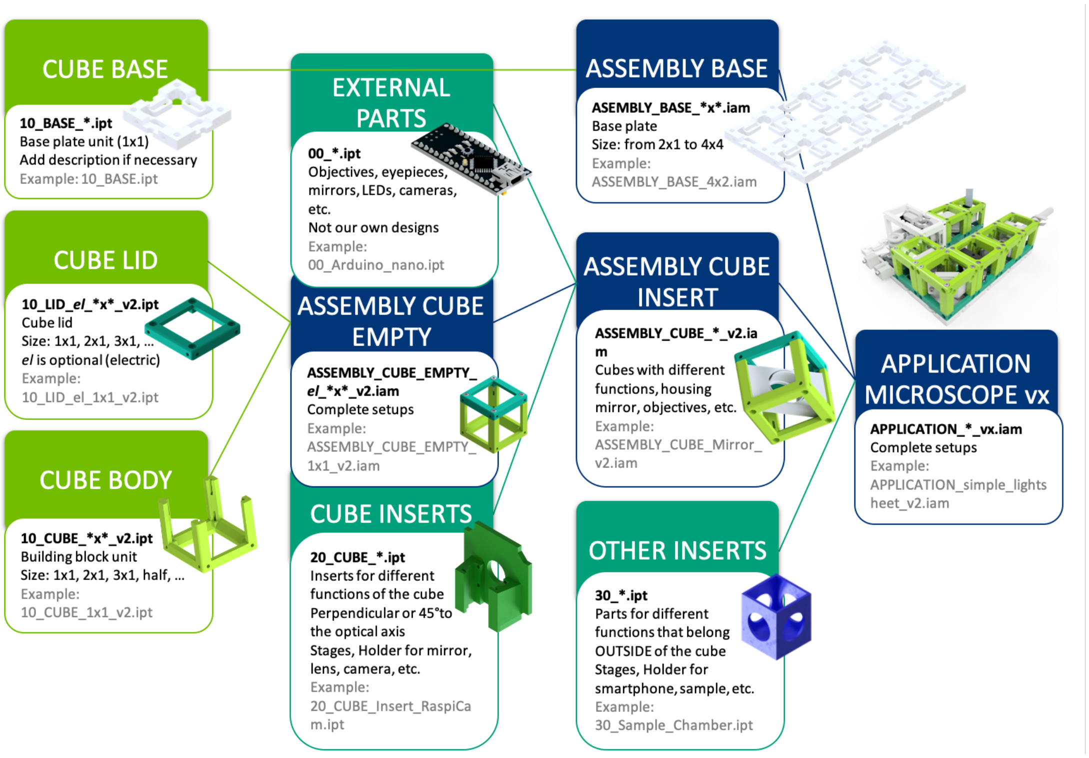

Figure 4. The chart showing logical structure of building a UC2 setup.

Table 1. Description for UC2-related modules and components

**Setup Name**<br>**Name**	Price<br>**Description**
**CUBE BASE**	The units of the base are joined into the **ASSEMBLY BASE**-plate ("skeleton" of the setups). This is the frame and backplane of the UC2 project, determining the size and layout of the optical system. UC2 **MODULES** attach to the rectangular baseplate slots using ball-magnets and ferro-magnetic screws. Additionally it supports "smart" **CUBE**s with electrical power.
**ASSEMBLY BASE**	Multiple **CUBE BASE** units can be screwed together or printed as a larger monolithic base-plate (e.g. 1×2, 4×4, etc). The base-plate has wholes which fits common optical table formates with a grid-spacing of $50 \times 50\,mm$.
**CUBE BODY**	The **ASSEMBLY CUBE EMPTY** consist of the **CUBE BODY**- and the **CUBE LID**-part which gets screwed together with M3 ferromagnetic screws. Screws can be inserted on all sides in order to build setups in three dimensions.
**CUBE LID**	The lid closes the **CUBE BODY** when attached by screw to the **CUBE BODY**. It can carry electronics like microcontrollers (e.g. Arduino, ESP32). By adding wires to the screws closing the **CUBE LID** electronic components can be supplied with electrical power.
**ASSEMBLY CUBE EMPTY**	The raw cube/basic building block is made of the **CUBE BODY** and the **CUBE LID** and can vary in size (e.g. 1×1, 1×2, etc). Note that the MDK only details the specification of the **CUBE** and **CUBE BASE** to the extent that it is necessary for module developers to develop modules.
**CUBE INSERT**	**CUBE INSERT**s are physical components that implement various functions into the system by adapting 1×1 to the **ASSEMBLY CUBE INSERT**. They fit the inner dimensions of the **CUBE EMPTY**. There are two types of **CUBE INSERT**s: *perpendicular* (to the optical axis) and *diagonal*. They serve as holder for various components like lenses, mirrors, cameras, filters, and other components demanded by the application. Existing **CUBE INSERT**s can be adjusted to fit specific parts (i.e. lens diameters).
**EXTERNAL PARTS**	Everything which is not part of the UC2-system or cannot be 3D printed is termed **EXTERNAL PARTS**. This can be commercially available parts like objectives, lenses, LEDs, etc., but also 3D-printed parts from other projects (e.g. openflexure stage).
**ASSEMBLY CUBE INSERT**	This is the combination of the **ASSEMBLY CUBE EMPTY** and a **CUBE INSERT**. Since the **ASSEMBLY CUBE**s are the optical building blocks of a UC2 setup, adding features is accomplished by hardware plugins also called **CUBE INSERT**s.
**EXTERNAL MODULES**	Using **EXTERNAL MODULES** one adapts **EXTERNAL PARTS** that typically do not fit inside a cube but give function to it. This can be for example cellphones, stages projectors, etc. Customized hardware adapters interface with the **ASSEMBLY CUBE**.
**MODULES**	Entire functional **MODULES** can be swapped to the system (e.g. ISM-module, projector). They have the correct screws and dimensions to adapt to the magnets on the baseplate. They are fully independent, but need to provide the Fourier- and image-planes at the proper position, such that adjacent cubes can relay them.
**APPLICATION**	**APPLICATION**s are complete optical setups. They are composed of one or more **ASSEMBLY BASE** units and **MODULES** with different functions. The GitHub repository provides a list of basic optical systems which are also compiled into a ready-to-use list called "TheBOX".

2 The Cube

The cube is the basic building block of the UC2 framework. Its purpose is to create a bridge between the toolbox and any external component which fits inside. It has a unit-pitch of $50\,mm$, with a hole-to-hole distance of $40\,mm$ and can be extended on an integer (i.e. 1×1 Fig. 5, 2×1 Fig. 6 etc) grid in all directions. The external size is $49,8\,mm$ to incorporate imprecision of the printer. The centro-symmetrical cube is designed so that the beam is guided vertically and through the centre of the cube sides. The free space in the cube's interior is large enough to accommodate common optical lab-ware (e.g. 1" cage system from Thorlabs, Edmund optics, Qioptics etc) and other components using customized adapters. Ferromagnetic worm and flat-head screws (DIN ISO 912, M3×18mm, DIN ISO906, M3×5mm, galvanized) also used to hold the cube together, connect to magnetically $5\,mm$ ball magnets sitting in the baseplate.

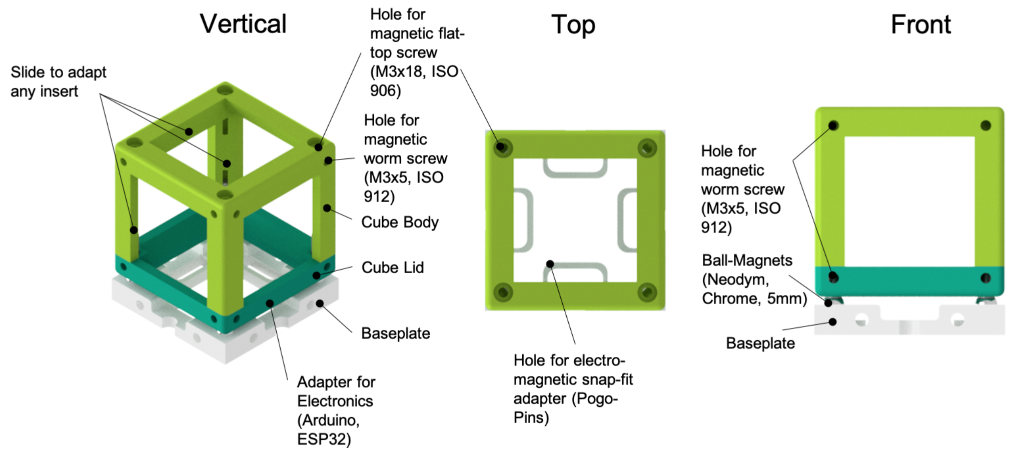

Figure 5. Basic empty cube 1×1 The basic cube consist of two parts, the frame and the lid which is hold together by a set of ferro-magnetic M3 screws. These screws attach to the ball-magnets inside the base-plate. The inner structure of the cube allows inserting a customized hardware plugin in all directions.

3 The Baseplate

The Baseplate (unit-size $50 \times 50\,mm$, magnet-to-magnet distance $40 \times 40\,mm$, Fig. 6) is the "skeleton" of the UC2 framework and holds the different modules in place and provides a straight optical axis. The neodymium ball-magnets are press-fit into the 3D printed baseplate thus creating a stable mechanical connection to the 3D printed cubes. Although the design is mechanically over-defined with its 4-point interface, it represents a compromise between a simple design process for optical assemblies and mechanical stability and versatility. The cubes allow convenient orthogonal alignment along an optical axis and are easier to stack compared to triangular pyramid or hexagonal units. Mechanical imprecisions e.g. due to faulty 3D printing can be compensated by adjusting the screws.
To provide electric components with power, wires added to the screws sitting in the cubes and to the conducting chromium ball-magnets can ensure an electric connection at a minimum number of visible cables since they are hidden inside the cube. In order to extend the grid in all room-directions the base-plate has holes at all faces to join multiple plates via screws together. Additional M6 holes enables adaption to optical tables, support boards or breadboards to assure stable and long-lasting mount.

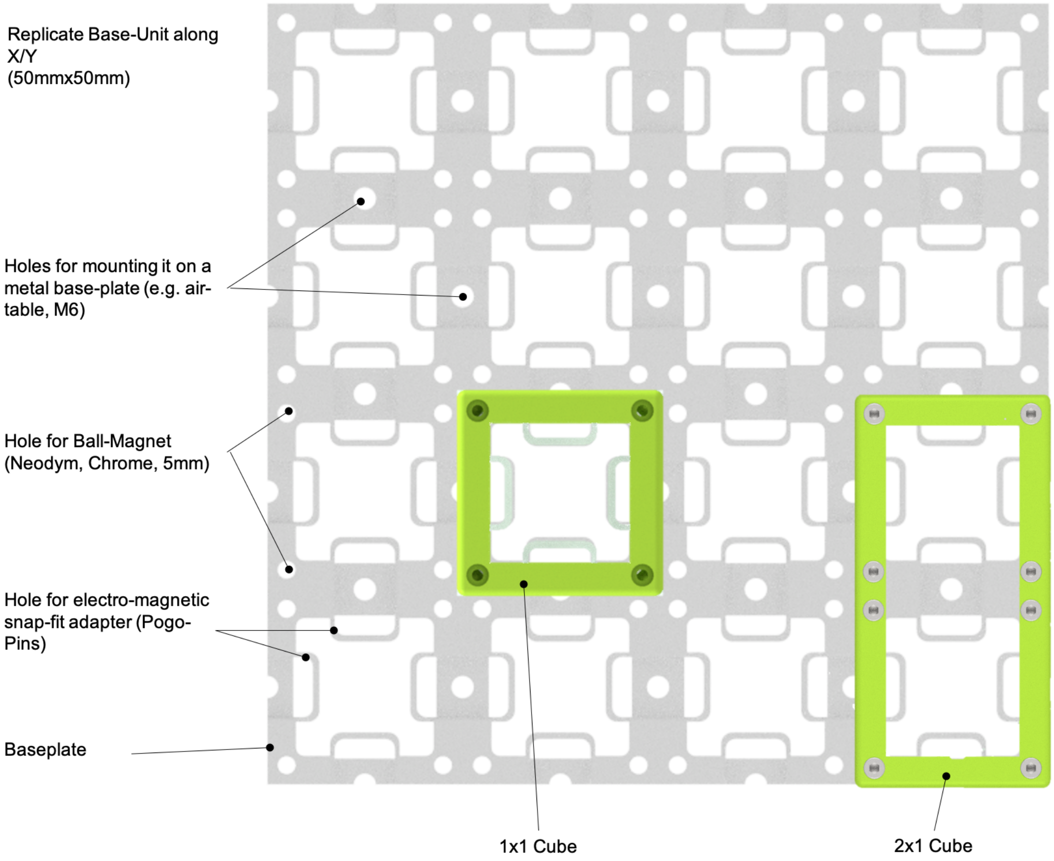

Figure 6. Baseplate 4×4 A exemplary assembly of a 4×4 baseplate equipped with two cubes. The M6 holes adapt to common optical tables to ensure long-lasting setups.

4 Cube Inserts

The cube inserts can be fully customized to adapt external elements, thus underlining the idea of creating an open-standard. The online-repository provides all relevant dimensions and CAD-Design templates for Autodesk Inventor and OpenSCAD to quick-start development with UC2. Additionally, a number of video-tutorials can be found in online video platforms. With this we invite people to develop their own modules and contribute with their designs to the UC2 system.

Inserts are slid into the cube which allows to adjust the position along the optical axis. By having dedicated rulers and spacers, one can make sure, that the insert is parallel to the cubes' face and optical dimensions can be reproduced. The two CAD-files below show examples for inserts at an angle of 0° and 45° w.r.t. the optical axis, which fits into the standard centro-symmetric 1×1-cube.

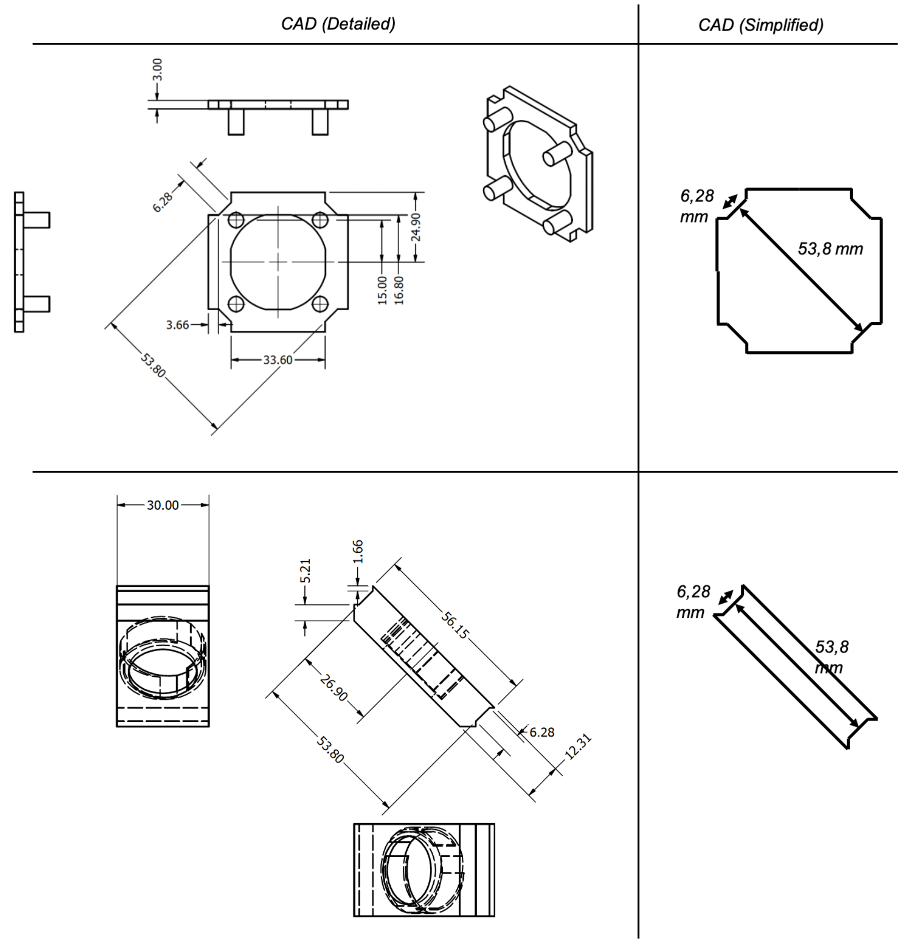

Figure 7. Generic design for a cube-insert Since the cube is centro-symmetric, an insert can be rotated in all directions. The figures show exemplary insert-designs for a 0°- and 45°-version, meant for a Thorlabs cage module and a mirror respectively. The smooth but slightly layer-structured 3D-printed surfaces allow an easy sliding mechanism, but keep components in a fixed position in the same time. Newly designed cubes-adapter or inserts simply need to follow the dimensions are visualized in the simplified version of the CAD drawing.

5 Good Practice to Transfer an Optical System to UC2

The core idea of the modularity inside the UC2 system is based around the Fourier-optical principle, meaning that adjacent lenses are placed so that focal planes of adjacent optical parts are coinciding in order to minimize effects like aberration and vignetting. This requires a focal-to-focal distance of multiples of $50\,mm$. Determining Fourier- and image planes as optical interfaces enables sub-grouping of the whole system into modules and optical building blocks. The optical axis always goes through the centre of and perpendicular to an open cube facet. Beam-folding by 90° in all directions (i.e. X, Y, Z) can be assured using mirrors.
In case of more complicated assemblies like the *open*ISM module, it is advisable to design a monolithically printed block to assure higher precision and robustness. The outgoing plane (i.e. image or Fourier plane) should again adapt to the following plane from the next cube/module.

A simple example is given by a Keppler telescope illustrated in Fig. 8 which can be accomplished by using two lenses ($f'_1 = 50\,mm$, $f'_2 = 100\,mm$) with a distance of $d_{1,2} = 150\,mm$ between their principle planes. The cellphone microscope shown in Fig. 8 gives another example how simple it is to create an imaging system, where the tube length of typical finite corrected objective lenses (e.g. $d_{tl} = 160\,mm$) is reproduced by the two folding mirrors and a spacer before the intermediate image gets relayed by the ocular and imaged by the cellphone camera.

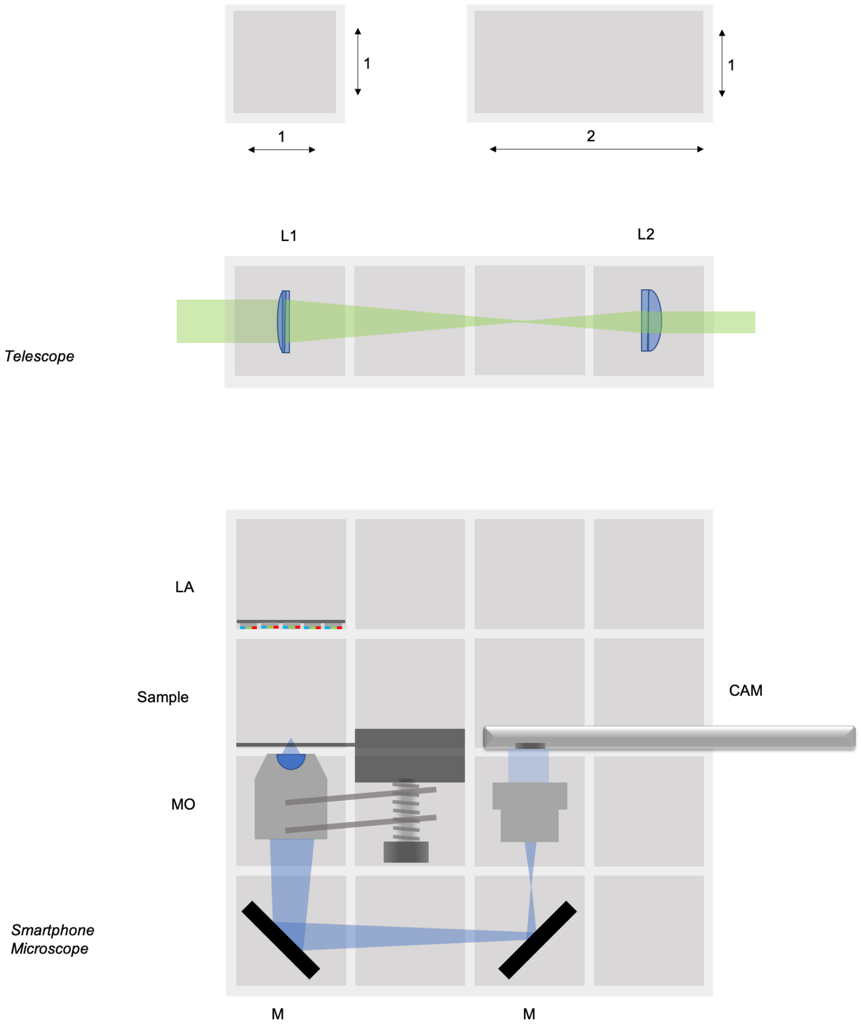

Figure 8. Good practice for UC2 assemblies - The core-units are the UC2 optical building blocks on a grid of integer $50\,mm$ (top). By combining two lenses $L_1, f'_1 = 100\,mm$ and $L_2, f'_2 = 50\,mm$ one can create a Keplerian telescope (middle); A more complex assembly can be created using objective lenses, LED matrices and oculars to create a smartphone microscope (below).

6 Software

6.1 Details about Hardware and Software Control

To create reproducible long-term measurements with the incubator-enclosed microscope, we created a Python-based GUI which runs on a Raspberry Pi equipped with a 7-inch touch-screen. A detailed description on how the system needs to be installed can be found in the dedicated software-repository. The user interface based on the kivy-framework (v1.11.0, [4]) is visualized in Fig. 9 and allows the control of several hardware elements such as individual addressing of LEDs in the LED-matrix, movement of motors connected to the system (e.g. along X, Y, Z) and intensity control of the fluorescent illumination. In addition to that, the software also allows the scheduling of long-time experiments. This includes the choice of the illumination modality (e.g. DPC, Fluorescence, bright-field, dark-field,etc), the timing of image capture and the overall duration of the experiment. The images captured using automatic settings such as auto-exposure and auto white balance (AWB), are saved as JPEG-compressed photos on the internal SD-card in order to save memory.

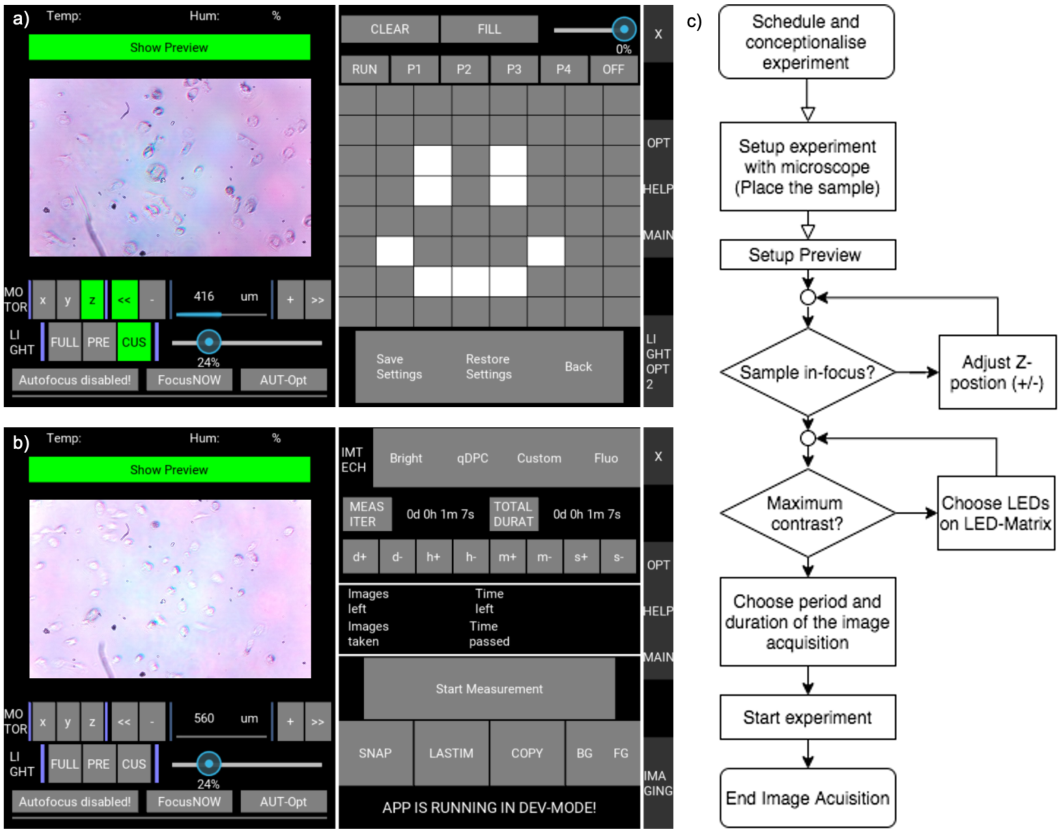

Figure 9. Basic settings for the GUI The GUI is divided in the hardware-control section a) and experiment configuration panel in which the user defines long term time lapse e.g. for the incubator-enclosed or light sheet microscope. c) an exemplary workflow of a typical biological experiment over multiple days is visualized.

The software can be used to control wired as well as wireless components connected to the Raspberry either via I^2C or WiFi. A dedicated UC2-I^2C-device adapter created in Python as shown in Fig. 10 preserves the modular nature of the UC2 system since the command-set

sent by the Raspberry Pi to any I^2C or MQTT device in the same network follows a modular instruction set.

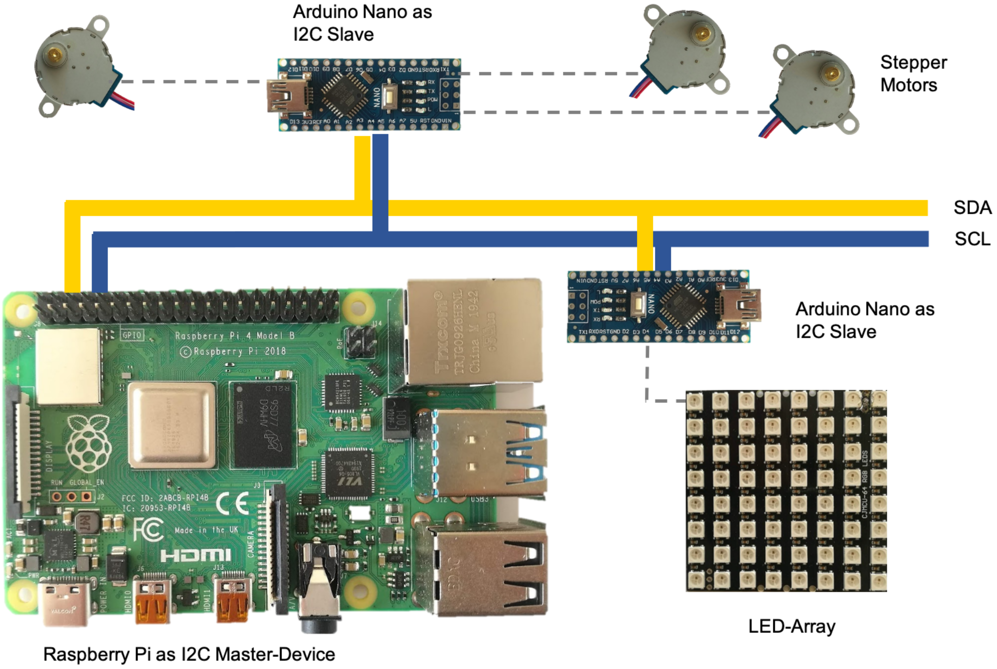

Figure 10. Schematics of the I^2C device adapter The Raspberry Pi acts as a I^2C master device which sends controlling commands to all slaves in the same network created by the four-wired signal (*5V:* power-signal, *GND:* ground-signal, *SDA:* signal data, *SCL:* signal clock). UC2 relies on low-cost Arduino Nanos which convert the I^2C commands into hardware control operations for motors, LEDs or anything else controllable through microcontrollers.

The MQTT-based wireless communication system visualized in Fig. 11 has the advantage that each device can control any other device. This is advantageous, for example, when one mobile phone is used as an image capture device and another mobile phone is used as a remote control for a setup. Since the devices can be reached from remote places through the internet, adjusting or readout of parameters could theoretically be done from any place which supports internet access.

Good practice for the TCP-IP based MQTT-network connection is to setup a dedicated WiFi-Router (Netgear Nighthawk R7000) which handles the different connections. A MQTT broker (i.e. server) can be created using either a cellphone or the Raspberry Pi by using open-source software such as Moquette[5] or Mosquitto[6]. We also developed a stand-alone Android application (APP) which incorporates the MQTT broker as well as the MQTT client in order to use the system independent from any external devices (e.g. in the field). The source-code can also be found in our software repository.

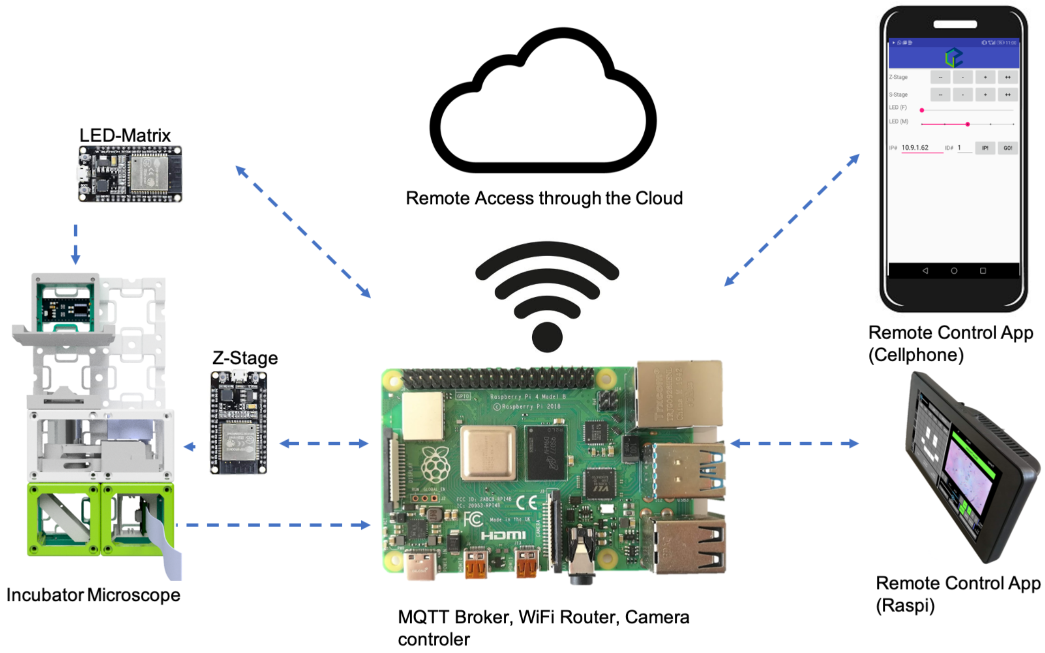

Figure 11. Schematics of the MQTT Connection All devices are connected to the same network (e.g. WiFi hotspot) and MQTT-broker (e.g. server) which can be represented by a Raspberry Pi. The MQTT-based network protocol allows multiple devices to be controlled remotely. Each MQTT client (e.g. ESP32) reacts on a sent command.

7 Experimental Details

7.1 Long-Term In-Incubator Microscopy

The aim of this biological study was the long-term observation of macrophages in vitro under different environmental effects. Other than putting an incubator-enclosed on a microscope stage, we decided to place the whole microscope in a bench-top incubator-enclosed (Heraeus Instruments, Germany) which ensures suitable conditions for living organisms (e.g. Temperature, acidity control via CO_2-level). We formulate requirements for long-term biological imaging as follows:

- Time-lapse imaging at one-frame per minute over several days
- Optical resolution on subcellular level (e.g. $2 - 3 \mu m$)
- Autofocus capability to potentially compensate sample drift, if necessary
- Bright-field and fluorescent imaging of labelled cells (e.g. CellTracker green)
- Customized illumination settings to enhance the visible contrast of weakly scattering phase objects
- Autonomous operation over long-time periods
- Host standard microscope slides and 35 mm glass-bottom petri dishes

The resulting prototype which was created based on these requirements using the UC2-system is shown in Fig. 12 and in more detail in the online repository. It results a simple optical path derived from an inverted compound microscope with a finite-corrected objective lens (Generic

brand, 10×, NA=0.3) yielding in a theoretical resolution of 1.8 μm with a coherently illuminated sample (e.g. only one LED on the optical axis). In order to reduce the overall size of this device, we reduced the tube length (e.g. distance between the objective lens and the intermediate image plane/camera sensor) from 160 mm to ≈ 100 mm which results in a longer working distance and reduced effective magnification. The optical resolution using the Raspberry Pi camera (V2.1, Sony IMX 219, $d_{pixel} = 1.12\,\mu m$, Bayer-pattern, $t_{exp} = 100ms$, UK) gives $d_{min} < 2.3\,\mu m$ and an effective magnification of ≈ 7× were quantified by imaging a USAF chart as visualized in Fig. 13. To achieve multi-modal imaging, we used a 8 × 8 RGB LED array (Adafruit #1487), where only a subset of the available LEDs are within the NA of the detecting objective lens. The selection of the LEDs was done through a customized GUI on the Raspberry Pi while the visible contrast was maximized. For fluorescent illumination we decided to use a dark-field-like epi illumination. A module, sandwiched between the objective lens (e.g. Z-stage) and the sample, hosts a number of high-power LEDs sitting on a star-LED, while the resulting dark-field illumination blocks the zeroth-order which makes selecting the emission filter more cost-efficient, since only the residing thus weaker stray-light has to be filtered out [7].
All electric components are connected to a micro-controller which was an Arduino Nano in the wired (e.g. I^2C) and an ESP32 in the wireless (e.g. MQTT) control-mode. The magnetically fixed LED-matrix can be easily removed to gain space during exchange of cell culture media or in case malfunctioned hardware needs to be replaced. Being free in the choice of the distance between the sample and the illumination unit provides additional space for wires and tubes for applications like flow-cytometry or lab-on-a-chip.

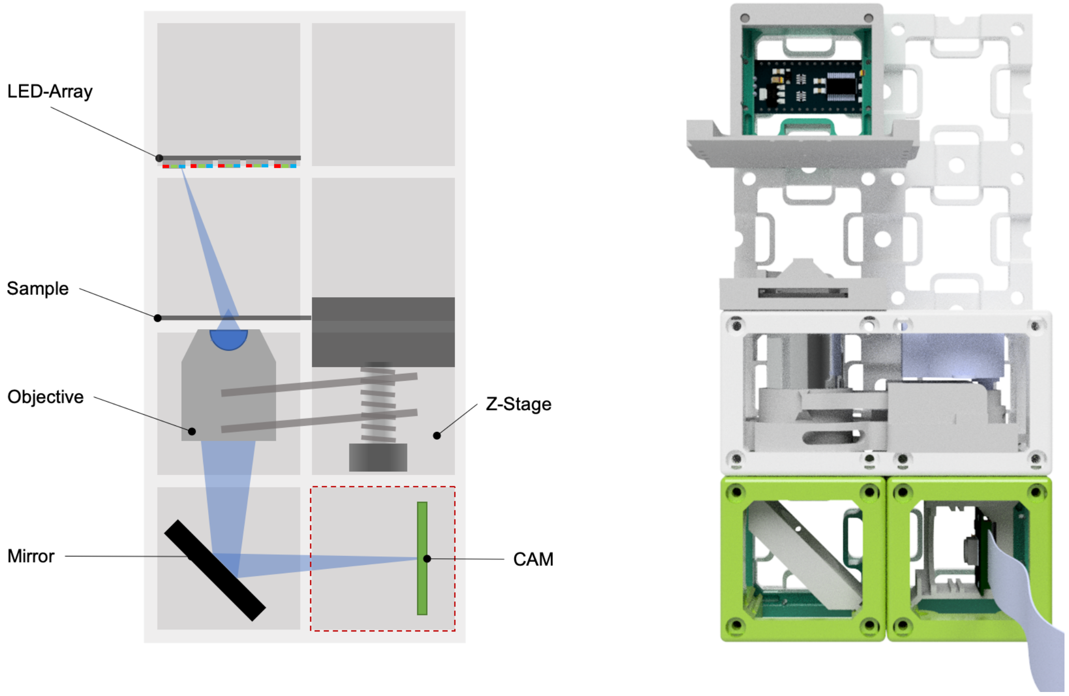

Figure 12. Scheme of the inverted microscope used in the incubator-enclosed - A LED-array allows the selection of the illumination angle and enables quantitative phase imaging (QPI). The objective lens inside the Z-stage can be moved up- and down by a stepper-motor controlled by an ESP32, while the optical path is folded using a simple mirror to form an image on the Raspberry Pi camera. The camera is connected to a Raspberry Pi equipped with a 7 inch touch-screen at an overall price-tag of ≈ 300 Euro

To be able to focus the sample during the acquisition series we designed a customized

monolithic Z-stage (see our GitHub repository) which is inspired by the open-flexure design of Bowman et al. [8]. It is based on a spiral-bearing where a level-arm pushes the objective up and down using a stepper-motor (China, 28BYJ-48). This way, the Z-stage produces no radial shift while it is moving. To ensure, that the sample stays in place, it is fixed by using a magnetic clamp, which simplifies its removal to replace the cell culture medium in the culture dish. The sample stage and Z-stage were printed using ABS to ensure sufficient thermal stability. Other components were made of PLA. An additional module which hosts a pair of low-cost stages allows XY positioning of the sample (see xyz-assembly) with a precision of $\approx 20\,\mu m$ which can be further optimized with micro-stepping. Yet, to keep things simple, we did not use this motorized XY sample stage in our experiments.

For in-vitro measurement, we placed the microscope into a standard S2 biological laboratory. We disinfected the microscope by spraying it with 70% ethanol. After setting up the microscope, the imaging parameters were selected and the microscope ran for several days before the data was transferred from the Raspberry Pi to an external storage medium for further processing. For the details on cell-preparation, see section 10.1.

The 8×8 RGB LED array also enables quantitative phase imaging based on the work by Tian&Waller[9], which is achieved by capturing a series of obliquely illuminated phase-objects and performing a deconvolution with the corresponding point spread functions. This feature was not used during the long-term acquisition since the contrast was sufficient and the additional effort to compute each result-image was rather high. To monitor long-term changes in the *in-vitro* experiments with low contrast cells (e.g. unlabelled macrophages) we relied on oblique illumination to exploit the phase gradient caused by the transparent cells.

All design files including the bill-of-material ($\approx$ 300 Euro)and an illustrated step-by-step assembly tutorial can be found here.

7.2 Optical Resolution, System and Long Term Stability

Since the very first experiment in the biological lab, the cube-based design went through a series of iterative optimizations, with components being exchanged and optimized over time. The portable design of the microscope simplified the transportation (e.g. using a bike, see Fig. 14) from the workshop to the University Clinics Jena (UKJ), where we performed the experiments. The iterative development process of the brightfield (BF) microscope required multiple design changes and exchanges of the modules which had to be transported from the optical laboratory to the UKJ every time. The approx. 6km distance was covered by bicycle, whereby strong vibrations did not affect the imaging quality and stability of the microscope in the field. We directly used the transportation as a stress-test of system-robustness by only roughly packing it into a bag and then carrying the light-weight systems by bicycle. Even after many transports, the systems are still taking images of identical quality.

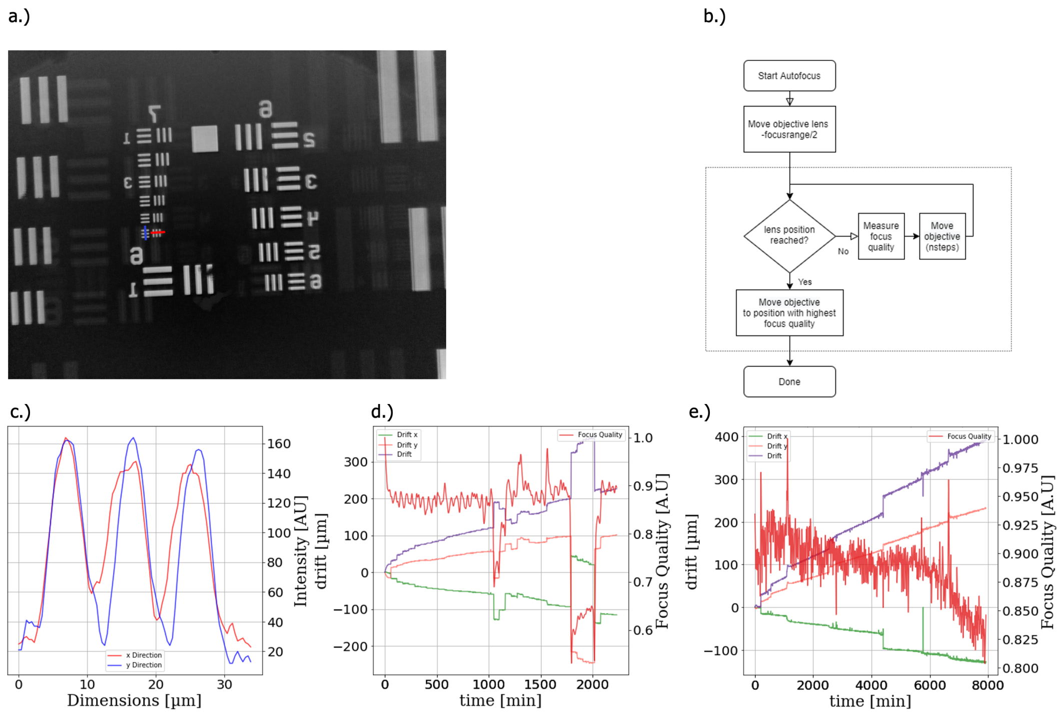

Figure 13. Calibration using the USAF target and autofocus algorithm - a)-b) The incubator-enclosed microscope can resolve subcellular features demonstrated by imaging Group 7/Element 6 ($4.4\,\mu m$/line pair of a USAF 1951 chart (Thorlabs, R3L1S4P, USA) yielding a resolution $d_{min} < 2.19\,\mu m$. c) An exemplary plot shows how the PLA printed Z-stage drifted over time with autofocus turned on, whereas in d) the autofocus was turned off. Discontinuities in the graph are due to door opening and loss of focus in the periodic autofocus routine. An almost linear drift in XYZ over time can observed.

Due to the use of 3D printed thermoplastic material (PLA, ABS), certain parts tend to bend during long-term experiments. By choosing ABS over PLA in places, where components experience larger tension, like the z-stage and the base-plate, the problem can successfully be compensated. We found, that once the Z-stage settled, it experiences almost no deformation over long time. One stage equipped with a $10\times$, $NA = 0.3$ objective lens was in focus even after 3 month, which can be appreciated during the 7-day measurement in Supp. Videos 1 and 2, where no automatic or manual refocusing was performed.
Even though we decided to use ABS for printing stages, which proved to yield sufficient long term stability in an incubator after the initial thermal equilibration period, we decided to test the thermal stability of PLA, which is easier to print, especially on low-budget printers. However, over time a deformation of the material can be observed, mainly caused by the heavy microscope objective lens. Even after adding additional supporting material, the objective pulled the Z-step mechanism downwards. In Fig. 13d) we show an exemplary drift plot in (X/Y/combined; green, orange, purple) over several days with the autofocus switched on (see next section for more information) in a bench top incubator-enclosed at $37°C$ and 100% humidity. An about $200\,\mu m$ can be observed over a measurement period of about 2 days. The slope is almost linear after the warm-up phase within the first 2 hours. A comparison with the same measurement with autofocus turned off leads to a constant drop of focus quality as indicated in Fig. 13e).

Although 3D printing gives the opportunity to build the system anywhere in the world, it is not yet on the optimal level of reproducibility. Two same printers will always give a slightly different result and the variation between the many brands of 3D printer can be significant. In order to compensate for this, one needs to iterate over many versions of the same design,

producing a lot of plastic waste. We found the method to be extremely useful for development but less beneficial in the production phase.

7.3 Autofocus for long-term in-focus measurements

For long-term measurements in biological laboratories, it was of great importance to obtain sharp images over long periods of time. This led to the development of a software-based autofocus algorithm that regularly refocuses the objective lens during experiments. The simple algorithm, as shown in figure 13b) performs a full scan along the Z-axis between a minimum and maximum position, maximizing the image sharpness: $argmax_z var(I(z) \otimes g)$, where $var(\cdot)$ indicates the variance in each intensity image at a Z-position $I(z)$. Low-pass filtering using a convolution with a Gaussian kernel g helps to remove noise that may result in unwanted high frequencies dominating over in-focus structures. Alternatively the direct spatial filter (i.e. Tenengrad) [10] image sharpness metric can be used.
The red path in fig. 13d) shows the focus quality over time. Every hour the microscopes refocussed, as seen by the periodic spike structure. From minute 1750 the autofocus lost its focus completely, which is also visible in the drift plot (violet). This was most probably caused by opening the incubator door. The system restored the focus after about two hours. Even though the autofocus routine worked to our satisfaction, it was finally not needed as the ABS material in combination with the relatively low NA provided sufficient stability for our long-term experiments.

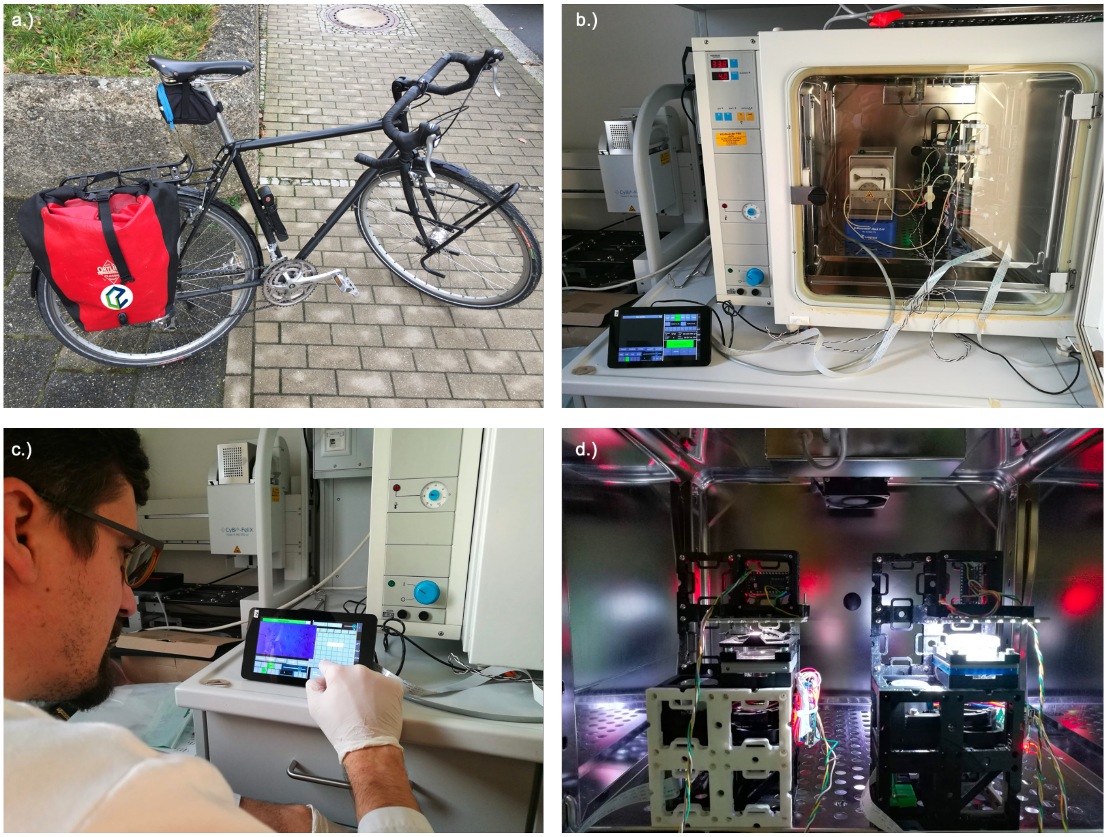

Figure 14. Setting up the incubator-enclosed microscope - a) The microscope fits inside a small box and can conveniently be printed and assembled and transported to the bio-lab using a commuter bag on a bike. In b) we show a customized application, where a microfluidic Ibidi µ-chip with endothelial/macrophage perfused co-culture was placed on the incubator-enclosed microscope, before all cables were connected. c) The next step requires setting up experimental details such as duration and interval, as well as illumination settings using the touch-screen on the GUI. d) due to their small footprint multiple devices fit inside a single incubator for multiplexed experiments.

Especially in long-term experiments it is of great importance that environmental vibrations are minimized. The bench-top incubator (Heraeus Instruments, Germany) was placed on an ordinary lab-bench which experiences low-frequency vibrations resulting from footsteps. The fluctuations of the FOV in all long-term experiments (see Supplement 1) were reduced to an acceptable level using a heavy metal-plate as a base for the microscope during the experiments.

7.4 Optical resolution of fluorescent data using FRC

To provide additional information about the optical resolution of fluorescent imaging, we provide a benchmark between a cutting-edge research microscope (Zeiss Axiovert TV, Germany) with an emCCD camera (Andor iXon3 DU-897) equipped with an oil immersion objective (Zeiss, 100×, NA1.46, TIRF, Germany) and our UC2 fluorescence microscope based on infinity optics as indicated in Fig. 15. The setup in Fig. 15 uses a 635/637 nm entertainment laser (laserlands.net, #3450, Dot Laser Module, 300 mW, 50 Euro, China), expanded by a telescope cube and focussed by a lens ($f' = 180\,mm$, CGI-Versand, 10 Euro, Germany) into the BFP of an oil immersion objective (No Name, 100×, NA1.25, 50 Euro, China) to produce an uniform illumination in the sample plane. The emitted signal is passed through a 50/50 beam splitter (optik-baukasten.de, 20 Euro, Germany), filtered by an emission filter (Chroma GQ6750/50, 200 Euro, Germany)

before a tube lens ($f' = 180\,mm$, CGI-Versand, 10 Euro, Germany) formed an intermediate image. An adjacent eyepiece (10×, Leitz Wetzlar, 10 Euro, Germany) maintained proper imaging condition for the cellphone and Raspberry Pi camera, both equipped with an objective lens. This means, that the exit pupil of the eyepiece (i.e. Ramsden disk) matches the entrance pupil of the objective lens.

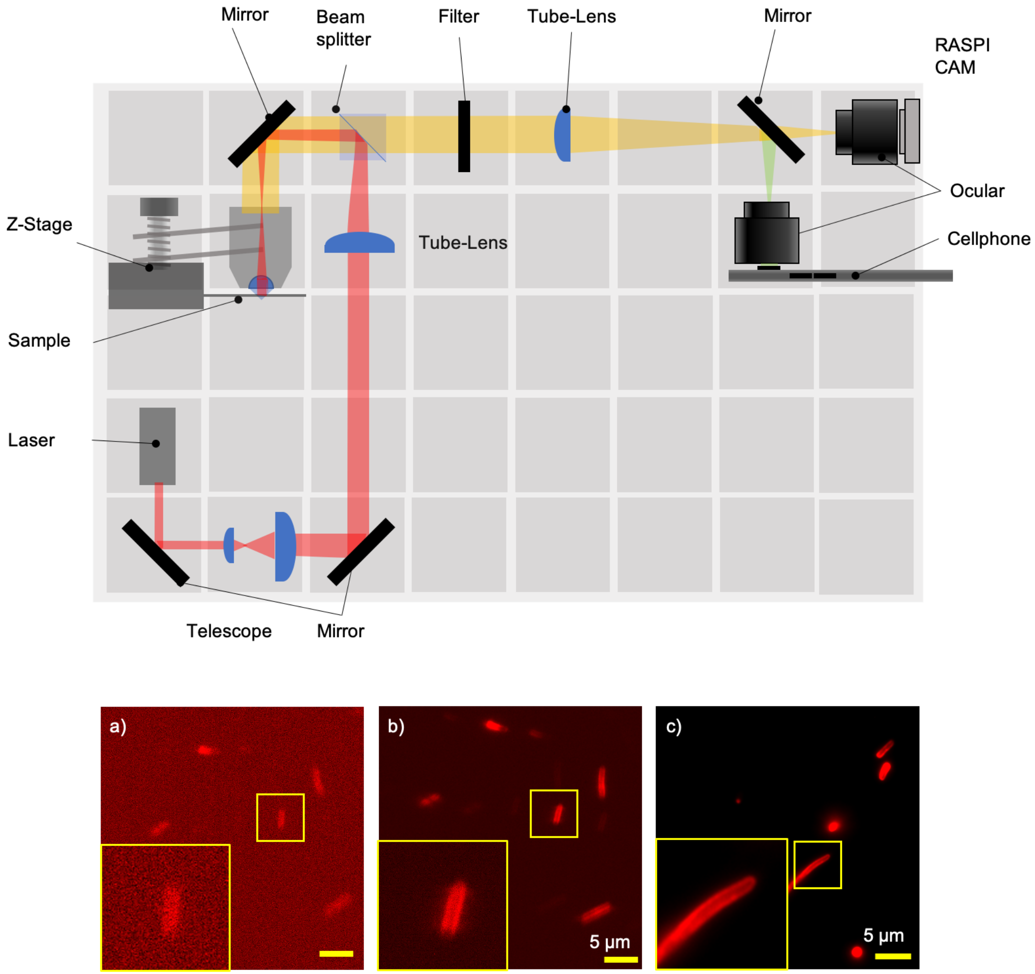

Figure 15. Fluorescence microscope based on infinity optics - The setup shows the arrangement of modules according to a typical inverted microscope equipped with infinity optics. Therefore a laser (635/637 nm) is expanded and focused into the BFP of the objective lens. The resulting plane wave excites the fluorescently (mCLING ATTO647n) labelled sample (*E. Coli*). Using the UC2-based setup the Raspberry Pi a) and cellphone camera b) are compared to a research-grade microscope c) (Zeiss Axiovert TV) equipped with an emCCD camera (ANDOR iXon3 DU-897). The improved SNR in case of the monochromatic cellphone camera sensor clearly resolves the bacteria membrane, which cannot be seen in case of the Raspberry Pi camera.

As a testing sample we rely on ATTO647N (SYSY, Germany) labelled *E. Coli* fixed on a coverslip (for protocol see Supp. Notes 10.6). The qualitative comparison in Fig. 15a)-c) between the inverted research microscope and the UC2 setup (see Fig. 15) shows an increased noise level

in case of the Raspberry Pi camera Fig. 15a) which results in a loss of fine structures, like the bacterial membrane which is clearly visible in case of the cellphone camera in Fig. 15b). We relied on RAW-frame acquisition to avoid unwanted artifacts due to denoising, background-level subtraction or compression. In case of the Raspberry Pi, we used a custom-written Python program based on the *picamera* library (v1.13), which saves the RAW Bayer pattern, where we extracted the red-channel. Similarly, we used a custom-written Android application, which captures unprocessed RAW frames from the Huawei P20 monochromatic camera available under https://GitHub.com/bionanoimaging/cellSTORM-ANDROID. We set similar acquisition parameters in both experiments, being $t_{exp} = 6.6\,ms$ and $ISO = 800$ in case of the Raspberry Pi and $ISO = 1000$ in case of the cellphone camera, since $ISO = 1000$ is not available in the Raspberry Pi.
Performing a Fourier ring correlation (FRC, [11]) in Fiji [12] (1.53c) using the BIOP Toolbox (BioImaging & Optics Platform, EPFL, Switzerland), yielded a resolution (see Tab. 7.4) of $d_{cellphone} = 0.6\,\mu m$ and $d_{Raspi} = 1.13\,\mu m$ compared to the Zeiss microscope, exhibiting a resolution of $d_{Zeiss} = 0.27\,\mu m$.

Setup	Camera	Objective	Resolution
UC2 fluorescence microscope	Huawei P20 Pro (monochrome, RAW, with lens)	100×, NA1.25 oil	$0.597\,\mu m$
UC2 fluorescence microscope	Raspberry Pi, Camera v2 (RAW, with lens)	100×, NA1.25 oil	$1.127\,\mu m$
Zeiss Axiovert TV	Andor iXon3 DU-897	Zeiss 100×, NA1.46, TIRF	$0.267\,\mu m$

7.5 Compare read noise between Raspberry Pi and Huawei P20 Pro camera

To further characterize the two different back-illuminated CMOS camera sensors of the Raspberry Pi camera v2 (Sony IMX 214) and Huawei P20 Pro monochrome camera (Sony IMX268), we performed a read noise calibration. To this aim, we acquired a temporal stack of 10 dark and 10 bright frames, which covering ideally the full available dynamic range. A customized python program, based on the NanoimagingPack Toolbox available under //https://test.pypi.org/project/NanoImagingPack/, computed the background, gain and read noise level [13, 14]. We performed this for different image acquisition parameters such as varying gain (ISO) and exposure time (t_{exp}), summarized in Tab. 7.5.

**ISO**	**100**	**200**	**500**	**800**	**1000**	**3200**	**6400**
**Huawei P20 Pro:**							
t_{exp}(Huawei P20 Pro) $[ms]$	16.6	8	4		1	0.5	0.25
read noise $[e^{-}RMS]$	2.53	2.57	1.97		1.88	2.24	1.91
gain ($[e^{-}/adu]$)	2.75	1.63	0.68		0.37	0.16	0.07
**Raspberry Pi camera:**							
t_{exp}(Raspberry Pi Camera V2.1) $[ms]$	20	10	4	2			
read noise $[e^{-}RMS]$	5.90	4.41	2.42	2.07			
gain ($[e^{-}/adu]$)	3.45	1.84	0.75	0.60			

Figure 16 shows an exemplary plot generated by the *cal_readnoise* routine of the NanoimagingPack applied to a) cellphone and b) Raspberry Pi camera images. The monochrome sensor showed a significantly lower read noise level. In both cameras, a linear dependence of the variance on the mean was observed, as one would expect from scientific grade sensor.

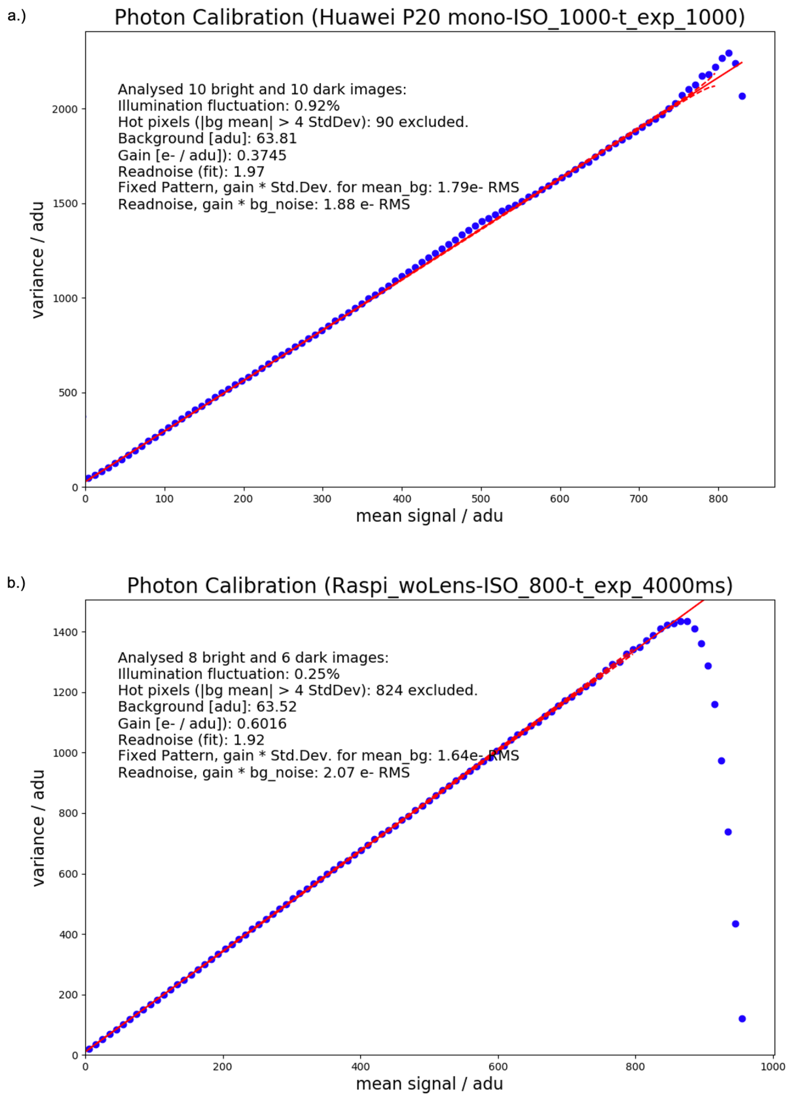

Figure 16. Read noise calibration of the Huawei P20 PRo and Raspberry Pi camera - a) Mean-variance plot generated using a series of unprocessed raw images acquired by the Huawei P20 Pro camera (ISO = 1000) and b) the Raspberry Pi camera module V2.1 (ISO = 800). In both cases a linear relationship between the variance per mean signal was observed. The monochromatic camera shows a significantly lower read noise level ($1.88\,e^{-}RMS$), compared to the Raspberry Pi camera ($2.07\,e^{-}RMS$) but both figures are comparable to scientific grade instruments even at lower gain.

7.6 Quantitative Phase: aIDT

To exemplify that our modular optical system can be used with a variety of different open-source image processing algorithms, we choose the freely available code for the annular intensity diffraction tomography *aIDT* from Li et al. [15]. The algorithm is especially interesting since it only requires a series of images with a varying illumination direction k_{illu}, and the algorithm can self-calibrate the illumination direction - ideal for a system which may experience slight misalignment over time. Other than methods like Fourier Ptychography Microscopy (FPM), the detection requires only illumination angles close to the edge of the detection pupil (i.e. close to dark-field illumination). We added an RGB LED-ring (Adafruit, #1643), where each LED can be addressed individually using a microcontroller (e.g. Arduino Nano, Espressif ESP32). We used only the green-channel to produce quasi-monochromatic light and acquired a set of images of fixed endothelial cells using a cellphone (Huawei P20 Pro, BI-CMOS Sony, IMX 286, China). It was of great importance to acquire the data in RAW-mode since the automatic calibration routine of the *aIDT*-algorithm failed when the images were compressed (e.g. JPEG).

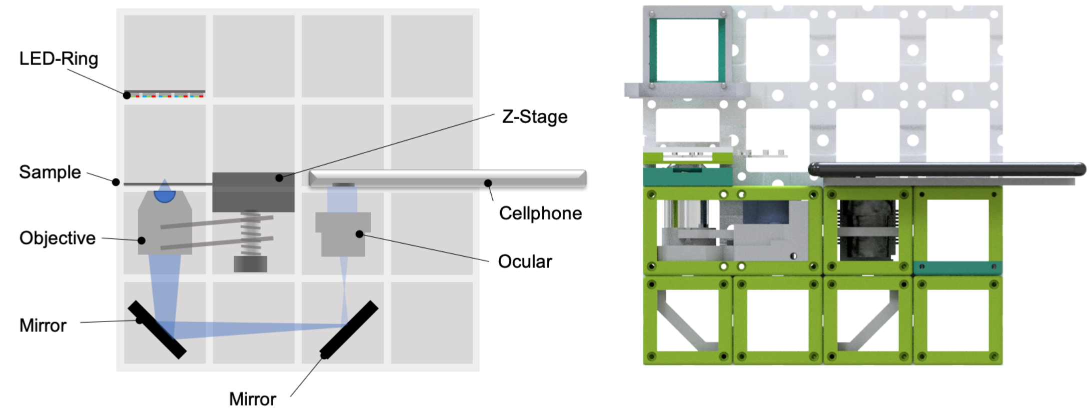

Figure 17. Scheme of the aIDT assembly using an LED ring and cellphone camera - The LED ring illuminated the phase sample from 16 different angles, which produces a series of images feeding the inverse model. This algorithm can recover a focus-stack of the quantitative phase. The cellphone can send MQTT commands to the LED-ring to synchronize illumination $\rightleftarrows$ frame acquisition.

The optical setup agreed to the incubator-enclosed microscope described in section 7.1, where we used a 10×, $NA = 0.3$ objective lens. The illumination NA had to be slightly less than the detection NA to position all illuminating plane waves inside the detection pupil which follows into the requirement of $NA_{illu} \leq NA_{det}$. Since the LED-ring has a radius of $r_{ring} = 16\,mm$, the NA_{illu} is governed by the distance between the LED and the sample d_{sample}:

$$NA_{illu} = tan(r_{ring}/d_{sample}) \quad (1)$$

$$d_{sample} = r_{ring}/atan(NA_{illu}) \quad (2)$$

which requires a distance of $d_{sample} \geq 54\,mm$ and was adjusted experimentally to about $74\,mm$, a smaller effective NA of illumination. To this aim, an additional layer in the base-plate (not shown in Fig. 17) was added. A modified version of the original code along with all necessary design files and manuals for this experiments are published in the GitHub repository.

7.7 Light Sheet Microscope

Even though the term "Ultramicroscopy" [16] has been around for almost a century, the topic selective plane illumination microscope (SPIM) or light sheet microscopy, where a thin light

plane illuminates a (fluorescently labelled) sample perpendicularly to the detection direction, gained lots of attention during the last decade. It provides gentle 3D-imaging of volumetric *in-vivo* and *ex-vivo* samples [17, 18]. Though this concept of optical sectioning in order to increase the optical resolution along the detection axis is straightforward, it becomes even more obvious if one experiences it in a hands-on experiment. Therefore, we started a series of workshops to demonstrate the working principle of these microscopes to their users, available with a comprehensive alignment tutorial in online repository. The overall *open*SPIM-inspired setup visualized in Fig. 18 is kept simple in order to give users the chance to build these setups on their own. This simple configuration proved itself to be optimal for workshops. To improve the imaging quality, an eyepiece and a smartphone can be used for image acquisition.

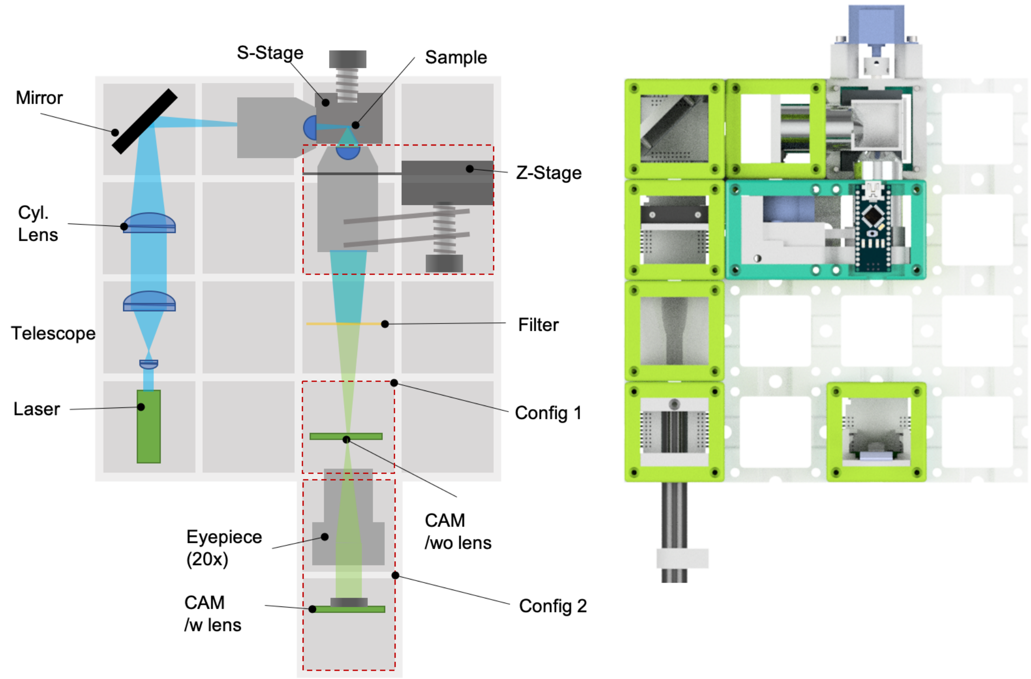

Figure 18. Scheme of the simple selective plane illumination setup - The left schematics shows the optical diagram of SPIM, where a laser pointer is first expanded and then shaped by a cylindrical lens and focussed by the illuminating objective into the sample. The detection is accomplished with a finite-corrected microscope perpendicular to the illumination plane (e.g. light sheet). In configuration 1 we show how the bare Raspberry Pi camera sensor (without the lens) can be used as the image detector. In configuration 2, an eyepiece relays the intermediate image formed by the finite corrected objective lens such that the exit pupil of the system better matches the input pupil of the Raspberry Pi camera (with the objective lens). It offers a larger FOV and better SNR since the camera captures more signal. The sample is placed on a Z-stage which can perform a focus stack of samples placed in a water chamber or on a slide. A kinematic mirror mount can be used to align the light sheet.

7.7.1 Optical setup

The setup hosts a blue laser pointer ($\lambda_c = 445\,nm$) as the illumination source, which gets expanded by a telescope. This telescope first focusses the incoming parallel light using a cellphone lens (Apple, iPhone 5, $NA = 0.24$, $f' = 3.2\,mm$, $\approx$ 5Euro) before being collimated by a second lens ($f' = 20\,mm$) to achieve a magnification of $\approx 6\times$. This beam gets shaped by a cylindrical lens (Comar optics, $f' = 63\,mm$) to create the 1D line-profile before it passes a kinematic mirror

mount cube featuring ball-magnets sitting on 3 ferromagnetic M3 screws, followed by a magnetic plate (e.g. galvanized steel, $30 \times 40\,mm$). The light sheet is further focussed by the illuminating objective (e.g. $4\times$, $NA = 0.14$) into the sample. The resulting light sheet inside the sample plane has a theoretical thickness of $200\,\mu m$ based on rather pessimistic assumptions on the profile of the laser-diode, while the actually measured thickness is around $50\,\mu m$ and thus slightly better than the depth of field (DOF) of the objective lens $d_z \approx 60\,\mu m$. When using a static light sheet, aligned onto the in-focus plane of the detection path, the Z-stack is obtained by moving the stage that carries the sample and acquiring an image for each step. The 3-dimensional image is then reconstructed.

The sheet illuminates the fluorescent sample sitting on a movable sample stage. The sample stage is equipped with a stepper motor (China, 28BYJ-48), which pushes a magnetic plate sitting on a flexure bearing. The step size is governed by the pitch of the screw and the smallest step size of the motor which leads to a reproducible step size of $\approx 25\,\mu m$. The sample holder, which can accommodate syringes with samples embedded in agarose, is equipped with 3 ball magnets to position the sample coarsely such that the sample is in focus of the imaging objective lens. Additionally, a 3D-printed water chamber can be placed on the moving sample stage to reduce scattering and aberration of the illuminating as well as the detection beam path.

The detection path (Fig. 18, green) follows a typical finite-corrected microscope scheme as also illustrated in Supplementary 7.6, where either a $4\times$, $NA = 0.14$ or a $10\times$, $NA = 0.3$ objective lens was used. As detector we choose either the Raspberry Pi camera module (V2.1) without a lens (Configuration 1 in Fig. 18) or a Raspberry Pi/cellphone camera with a lens but combined with an eyepiece (No Name, $20\times$, China) (Configuration 2 in Fig. 18). For all volumetric images presented in this manuscript, a Raspi camera equipped with the objective lens together with the eyepiece were chosen. As an emission filter we relied on a gel-filter (Lee, #010, medium yellow). Z-stacks can be conveniently acquired using the GUI running on the Raspberry Pi. It automatically moves the sample step by step and acquires an image for N Z-positions.

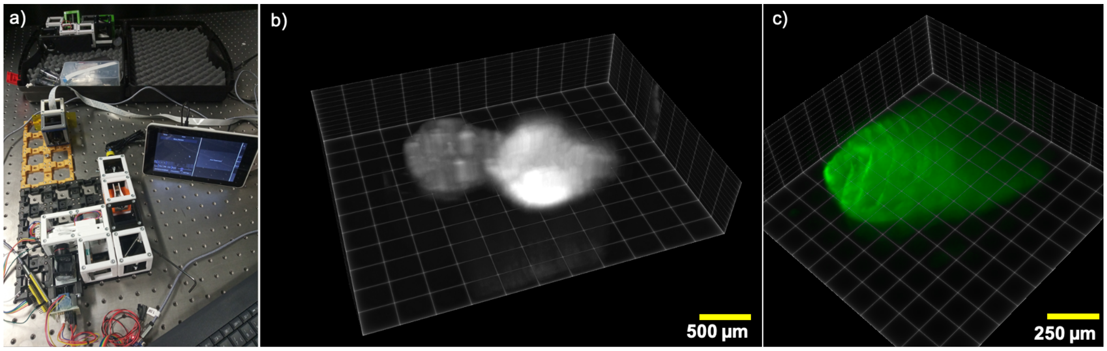

Figure 19. Light sheet microscope a) Complete light sheet setup with an eyepiece in front of the Raspberry Pi camera to increase the FOV. b) 3D reconstruction of a zebra fish embryo head and c) 3D reconstruction of a drosophila larvae from a z-stack obtained with this setup. The rendering was performed in Clearvolume [10].

7.7.2 Alignment of the setup

We provide a detailed description of the alignment procedure in our GitHub repository. Additionally the Video Supplement 5 gives an introduction on how to convert the incubator-enclosed into a light sheet microscope within 5 minutes.

7.8 Conversion from UC2 Incubator to Lightsheet Microscope

We assume all parts inside the FullBOX are already assembled and work properly. For each part we give a detailed build and assembly instruction in our GitHub repository. In the following paragraphs we will call the incubator microscope **IM** and light sheet microscope without the eyepiece (e.g. Raspberry Pi camera with removed objective lens) **LM**.

Table 2. Parts list for the **IM**

**Setup Name**	Position
Module	Position
Z-stage	IM 1
Raspberry Pi Camera Module	IM 2
Folding Mirror	IM 3
LED matrix	IM 4

Table 3. Parts list for the **LM**

**Setup Name**	Position
Module	Position
Laser	IM 1
Beam Expander (Telescope)	LM 2
Cylindrical Lens	LM 3
Kinematic Mirror (45°)	LM 4
Illumination objective (4×)	LM 5
Sample-stage	LM 6
Z-stage	LM 7
Raspberry Pi Camera Module	LM 8

7.8.1 Conversion Recipe: IM → LM

1. Prepare the textbfIM and the rest of the FullBOX
2. Place the 4×4 baseplate on an optical table, breadboard or other type of board
3. *optional:* Fix it using at least one M6 screw (for optical breadboard)
4. Take the UC2 laser module and place it in position **LM1**
5. Take the UC2 beam expander module and place it in position **LM2**
6. Take the UC2 cylindrical lens module and place it in position **LM3** and make sure the lens curvature is mounted perpendicular to the table surface
7. Take the UC2 kinematic mirror module and place it in position **LM4**
8. Take the UC2 objective lens module and place it in position **LM5**
9. Take the UC2 sample stage module and place it in position **LM6**. The motor has to be pointing away from the setup as shown in Fig. 20.
10. Take the Z-stage from position **IM1** and remove the fluorescent unit by unscrewing it. Then, move the objective coarsely in the direction towards the sample and place the stage in position **LM7**
11. Take the Raspberry Pi camera module from position **IM2**, attach the filter and place it in position **LM8**

12. Follow the detailed light sheet alignment tutorial for the **LM** in our GitHub repository to align the light sheet

13. Start the UC2 Incubator APP on the Raspberry Pi, which can be found in the Software GitHub and acquire Z-stacks by using the *Tomo*-mode

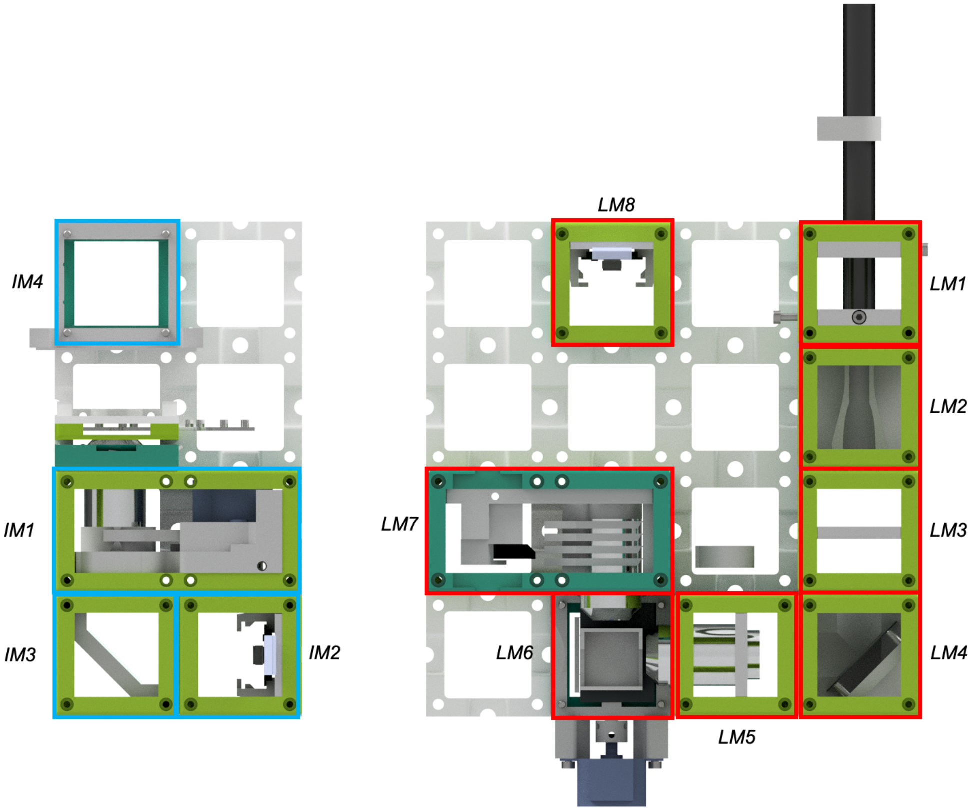

Figure 20. Conversion Process In order to build the light sheet microscope (right) using UC2 components, one can reuse several parts from the incubator microscope (left). All other parts can be found in the "FullBOX".

7.9 Image Scanning Microscope (*open*ISM)

Many consumer-grade electronics like video-projectors (e.g. digital mirror devices, DMD) or movie screens enabled entertainment "on-the-go" for a very low price, compared to scientific instruments, due to mass production. Besides wide-field projection systems based on liquid crystals on silica (LCoS), liquid crystal display (LCD) or DMD displays, more exotic laser-scanning-based systems (e.g. Sony MP.CL1A, Japan, $\approx$ 300 Euro) enabled us to create a UC2-ready image-scanning microscopes (*open*ISM) for around 300 Euro.
The laser scanner, equipped with a small Micro-Electro-Mechanical System (MEMS) scans a set of RGB ($\lambda_{blue} = 450nm$, $\lambda_{green} = 530\,nm$ and $\lambda_{red} = 650\,nm$) laser-beams over the 2D (e.g. X/Y) plane with a frame-rate of 60 fps at a spatial resolution of $1920 \times 720\,pixel^2$ to create an aerial image. A customized UC2 module enables the integration to our $50 \times 50\,mm^2$ standard. Following the work by Enderlein et al. (ISM) [19] and York et al. (iSIM)[20], we illuminate the sample with a nearly monochromatic, non-overlapping lateral grid of laser-light. The pattern then gets translated along a unit vector in X/Y such that the sum of all illumination-patterns corresponds to a laser-scanning bright-field illuminated image.

In Post-processing, all illumination spots (per frame) are treated in parallel. For each spot, a tile - meaning pinhole of multipixel size - is placed around its centre and gets extracted. Pixels at a distance to the nearest illumination spot then get displaced towards it by half its distance to account for the most-probable fluorophore position considering the current illumination and its detection position. Finally, the signal is integrated and written into the final image at the position where the tile-centre was placed. This procedure leads to a resolution of a factor up to $\sqrt{2}$ [21, 22] compared to standard confocal microscopy. The optical sectioning of this processing scheme is determined by the size of the virtual pinhole, the extracted region around each illumination position.

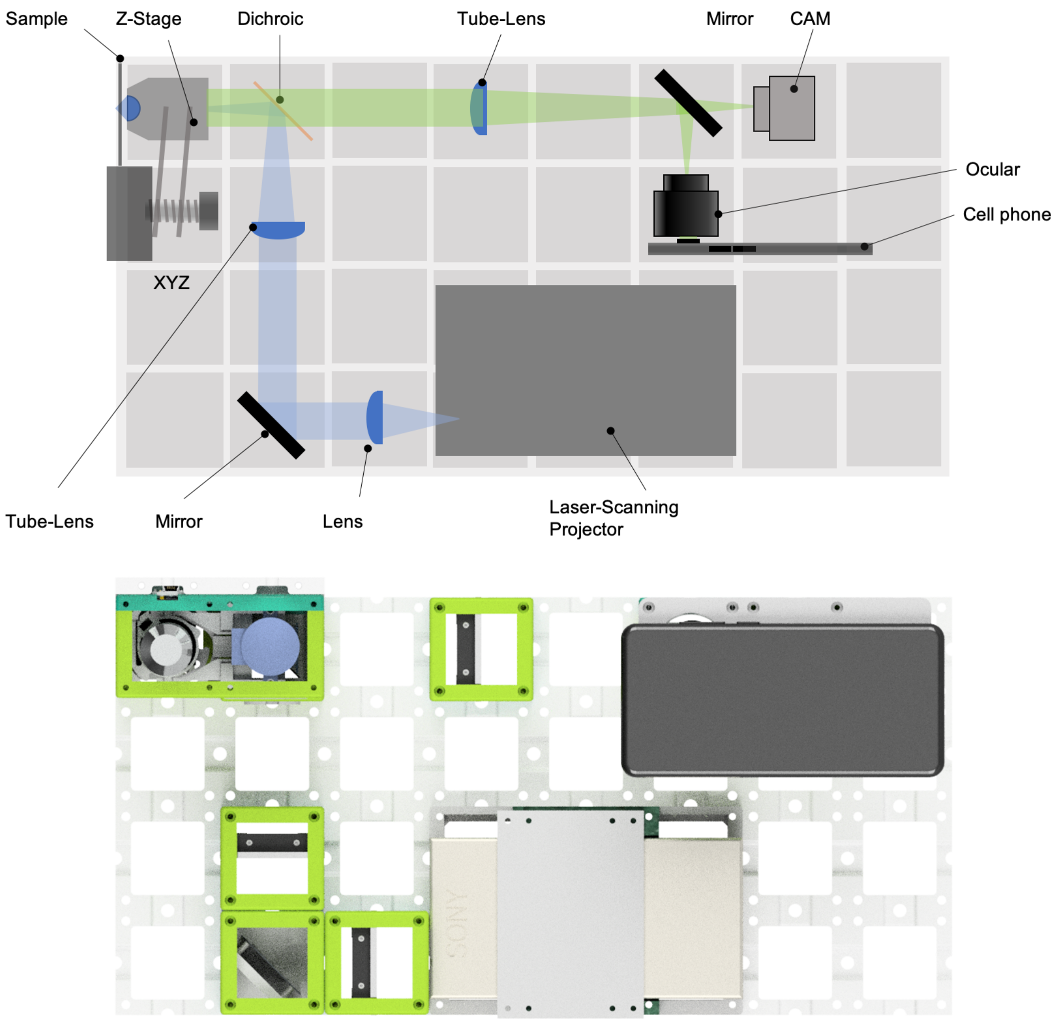

Figure 21. Scheme of the image scanning microscope (openISM) - The light path shown in the schematics above starts with the laser-scanning projector, where the beam gets collimated using the lens L_1 and re-imaged into the pupil of the objective lens using lens L_2. This telescope magnifies the mirror by a factor of 5. The detection path (green) follows a typical infinity-corrected microscope where either a CMOS (e.g. IDS, BASLER) or cellphone camera combined with an eye-piece.

All measurements in this manuscript are acquired using the monochromatic cellphone camera inside the Huawei P20 Pro. The design-files and additional explanation can be found here.

7.9.1 Optical setup and frame acquisition

The optical setup shown in Fig. 21 is straight forward. The resonating MEMS in the projector needs to be imaged into the pupil plane of the microscope objective lens. In order to get high-resolution images, the pupil is ideally over-filled by the image of the scan mirror. We assumed a diameter of the aluminium mirror of $d_{mirror} = 1.5\,mm$ and a pupil diameter of around $d_{pupil} = 5.5\,mm$ which requires a telescope, created by a lens $f_1' = 30\,mm$ and a following tube lens $f_2' = 180\,mm$. The low-cost infinity-corrected microscope objective (Optika, 20×, $NA = 0.4$, N-plan) was placed in a motorized Z-stage to allow focussing the objective relative to the sample. A set of different dichroimatic-mirror cubes with suitable filters (excitation/dichromatic/emission-filter: Comar Optics, 465 IK/510 IY/526 IB) allows the switching between different fluorophores and excitation wave-lengths. The detection path was generated by a $f_{TL}' = 180\,mm$ tube lens before a 20× mono ocular images to infinity. This way a cellphone-camera can create a sharp image if the focus is set to infinity. The effective pixel size depends on the selection of the cellphone and results in $d_{pix} \approx 150\,nm$ when using the Huawei P20 Pro.
Since the laser-scanner was not meant to be used for scientific instrumentation, technical details nave not been provided, making interaction with it cumbersome. Also, the uncommon pixel number of $1920 \times 720\,pixels^2$ leads to an unknown interpolation of the image provided by graphic cards, thus not resulting in "true" pixel information (i.e. one-to-one pixel relationship). We solved this by using a Macbook (Apple, 13-inch, MPXQ2D/A, USA) using an USB-C to HDMI adapter at a display-resolution of $720p$ in combination with a customized Python script which generates and displays the ISM-patterns. The monochromatic cellphone camera (Huawei P20 Pro, China) was driven using the open-source software FreeDCam ([23]), where the exposure time $t_{exp} = 1/60\,s$ matches the frame rate of the laser-scanning projector in order to reduce temporal bouncing effects of between frame rate and laser round trip.

7.10 Quantitative Imaging using *open*KOEHLER

An alternative to incoherent imaging methods, where fluorescently labelled cells are captured, is given by quantitative phase-imaging (QPI). This modality is very attractive for biological samples, because it is a label-free method thus obviating the time consuming labelling procedures which are sometimes also altering the behaviour or appearance of the subject of observation. Based on the adaptive illumination scheme described in [24], we incorporated a low-cost HDMI video-projector (40 Euro, Generic brand, China), which adds a fully adaptive illumination source to the system (see Fig. 22). The LCD-panel inside the projector was placed in a customized UC2 module, which includes the high-power LED for the illumination, the controlling PCB which translates the incoming HDMI video-signal for the 320 × 240 RGB 2,4 inch TFT screen (ILI9341, China) and a set of lenses to ensure correct Koehler illumination.
The LCD plane is a conjugated plane of the pupil plane of the microscopic objective lens and can create different illumination schemes such as oblique illumination, (quantitative) differential phase contrast (qDPC), Fourier Ptychography Microscopy, and Dark-field by addressing a specific bitmap pattern on the 2D plane. Each pixel produces a plane-wave in the sample-plane if it is in the "on"-state and can transfer a specific range of object frequencies. The (approximately) incoherent super-position of all (coherent) camera-plane images of the object describes the detected image according to the "Abbe"-method (see. [25]).

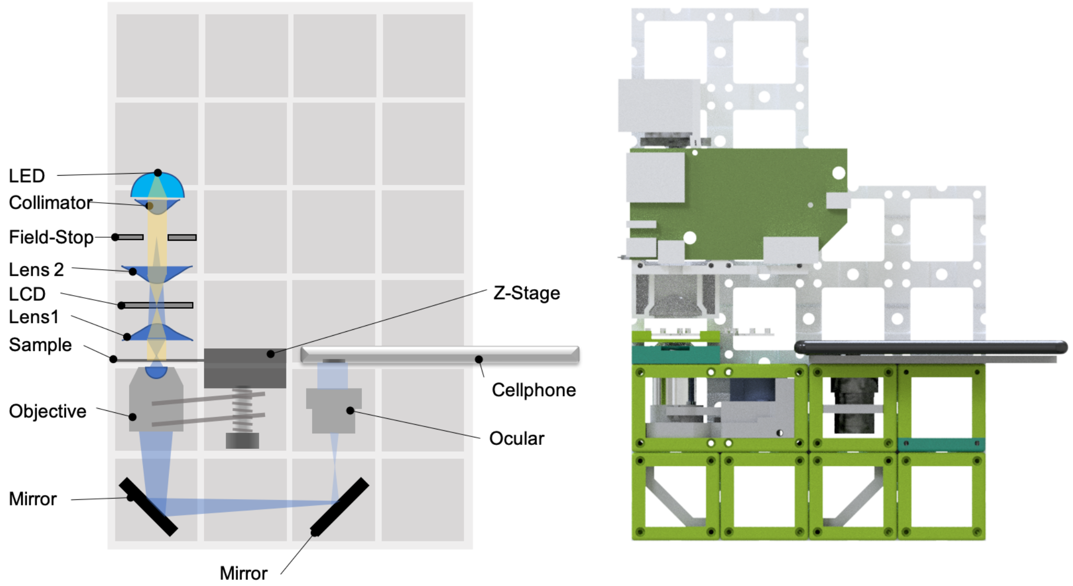

Figure 22. Ready-to-print *open*KOEHLER module - The *open*KOEHLER module accommodates an LCD optically conjugate to the objective pupil plane to create an adaptive illumination source. The module can be controlled using a standard computer equipped with an HDMI port. By varying the pattern in the LCD-plane, the contrast of transparent cells captured by a cellphone camera can be optimized.

7.10.1 Optical Setup

The optical system, shown in Fig. 22 follows Koehler illumination [25], where the condenser aperture plane is imaged into the pupil plane of the detecting objective and a field-stop is imaged into the sample plane to reduce stray-light from out-of-focus and regions. Taking the high-power white LED equipped with a collimating lens and adding two injection-moulded aspherical lenses (Thorlabs ACL3026U, $f' = 26\,mm$, $NA = 0.55$) L_1 and L_2 images the LCD-plane representing the aperture plane into the pupil plane of the objective and further images a field-stop in the sample plane.
A variation of the pattern displayed on the LCD controlled as a secondary display (e.g. HDMI-connection) directly influences the visible contrast. Depending on the displayed pattern (e.g. small point, disk, annulus, etc), the degree of coherence can be chosen freely. All files to replicate this experiment can be found here.

7.11 Educational Areas

The modular concept of building optical setups has proven to be very useful in demonstrations of various principles in microscopy and image formation in general. To exploit this potential, we designed *TheBOX*. The comprehensive toolbox provides components for explaining the basics of ray optics, diffraction and different microscopy modalities. It comes in two versions, *Simple* and *Full*. The *SimpleBOX* contains only passive components and covers the optical experiment of secondary and high school level. The *FullBOX* is equipped with electronics like microcontrollers (ESP32) and a Raspberry Pi microcomputer including a 7-inch touch-screen, keyboard and a camera module. In addition to basic experiments, this advanced box can create a compound microscope, a light sheet setup and other setups which are suitable for the everyday life in the biological lab. The complete list of setups can be found in the online repository. The target groups for *TheBOX* are schools and other institution that provide courses on optics and

microscopy. Thanks to its low price (600 Euro), a school/institution/course organiser can acquire or build multiple boxes and each participant can therefore have access to a hands-on experience, which is nowadays typically not the case. With this we try to provide the "Arduino for optics" meaning that the setup-time for getting started is heavily reduced by the plug-and-play nature of the optical building blocks.

We have successfully tested the concept of *TheBOX* in a series of workshops which are exemplary documented for the Inline Holographic Microscope and the Light sheet hackathon for the "International Day of Light" (IDOL) in the "Lichtwerkstatt Jena" and HHMI Research Institute on building a light sheet setup based on UC2 toolbox from scratch. *TheBOX* has proven to be a useful tool for microscopists, physicists, biologists and people generally interested in optics and microscopy at all skill-levels to learn e.g. the concept of Fourier optics or study organisms at a cellular level.

A set of trial-runs in Thuringian high schools (Carl-Zeiss-Gymnasium Jena, Montessori Schule Jena, Königin-Luise-Gymnasium Erfurt) to learn how the system can be used for "STEAM"-education (Science, Technology, Engineering, Art and Mathematics) yielded very positive feedback. This lead to interdisciplinary projects, where students study the application of the UC2 system to e.g. track micro-plastic in drinking water using special fluorescent markers. A series of tutorials on how to print, order and assemble can be found here.

7.11.1 The *"SimpleBOX"*

The *SimpleBOX* is a collection of optical experiments targeting elementary to high school level. It solely relies on passive optical elements such as lenses, mirrors and objective lenses. The overall material cost is in the range of 150 Euro from known online retailers. The various experiments are listed in the table 4.

Table 4. Overview of the experiments inside the *"SimpleBox"*

**Setup Name**	Description	**Link**
Keplerian Telescope	Telescope based on two positive lenses to magnify an image by a factor of 2, the image is up-side-down	GitHub-URL
Galilean Telescope	Telescope based on a positive and negative lens to magnify an image by a factor of 2, the image is upright	GitHub-URL
Projector	Projector based on a transparent object (e.g. slide) which gets imaged on a surface using a torch	GitHub-URL
Smartphone microscope	inverted microscope based on an objective lens, two mirrors and an eyepiece	GitHub-URL

Each setup comes along with a template, which shows how to assemble this optical module as shown in Fig. 3.

7.11.2 The *"FullBOX"*

[H] *FullBOX* is an extended version of the *SimpleBOX* which adds active components like motors, LEDs and electronics to the cubes making them "smart". Using micro computers like the Raspberry Pi and microcontrollers like the ESP32 or Arduino, can create more complex and fully autonomous setups ready for the every-day measurement in the optical lab or for the use in high schools and universities. The overall material cost is in the range of 600 Euro from known online retailers. A list of achievable experiments is given by table 4.

Table 5. Overview of the experiments inside the *"FullBOX"*

**Setup Name**	Description	**Link**
In-incubator microscope - transmission	Inverted microscope equipped with an LED array for bright-field microscopic imaging	GitHub-URL
In-incubator microscope - epifluorescence	Inverted microscope equipped with a dark-field-like LED illumination for fluorescent microscopic imaging	GitHub-URL
Light sheet microscope	Selective plane imaging using using a Raspberry Pi camera	GitHub-URL
Smartphone microscope	Inverted microscope equipped with an LED array for bright-field microscopic imaging using a cellphone camera	GitHub-URL
Abbe diffraction experiment	Experiment to observe Fourier-filtering by imaging the image and the Fourier plane simultaneously	GitHub-URL
Spectrometer	simple spectrometer based on a reflective grating from a CD/DVD	GitHub-URL

Using additional components like the scanning laser projector-based *open*ISM, the *FullBOX* can be extended to individual needs.

8 Bill of material

We provide a comprehensive bill-of-material for all assemblies, applications and boxes in an interactive spreadsheet. The prices heavily depend on the choice of the retailer and distributor and can be observed in Table 6.

Table 6. Cost-breakdown of the ready-to-use boxes and presented setups

**Setup Name**	Price
SimpleBOX	146, 81 Euro
CourseBOX	295, 22 Euro
FullBOX	618, 33 Euro
In-incubator microscope	314, 38 Euro
Light sheet microscope	423, 79 Euro
Smartphone microscope	134, 43 Euro

Below we will give an in-detail summary of all the components which were used in the incubator-contained and light sheet microscope.

Table 7. Light sheet microscope: Complete Bill of Materials

Module	Part	Printable	Quantity	Source	Unit price (Euro)
**ASSEMBLY_Baseplate 4×4**					32,68
	Baseplate 4×4	1	1	Link	2,6
	Ball magnets	0	64	Link	0,47
**ASSEMBLY_CUBE_empty_1×1**					2,2
	10_Cube_1x1	1	1	Link	0,4
	10_Lid_1x1	1	1	Link	0,2
	Screws M3×12	0	4	Link	0,2
**ASSEMBLY_CUBE_Beamexpander**					11,8
	ASSEMBLY_CUBE_empty_1×1		1		2,2
	20_Cube_Insert_Beamexpander	1	1	Link	0,3
	30_Lens_Adapter_Beamexpander	1	1	Link	0
	iPhone Lens	0	1	Link	4,6
	Planoconvex Lens f'=26,5mm, 18 mm (551.OAL)	0	1	Link	5
**ASSEMBLY_CUBE_Laser**					18,4
	ASSEMBLY_CUBE_empty_1×1		1		2,2
	20_Cube_Insert_Laser_Mount	1	2	Link	0,3
	00_Laser_Clamp_OnOffSwitch	1	1	Link	0
	Blue laser pointer, 445 nm	0	1	Link	14,8
	Screws M3×18	0	4	Link	0,2
**ASSEMBLY_CUBE_Lens_cylindrical**					62,4
	ASSEMBLY_CUBE_empty_1×1		1		2,2
	20_Cube_Insert_Lens_Cylindrical	1	1	Link	0,2
	Cylindrical lens, f' = 63 mm (63 YQ 40)	0	1	Link	60
**ASSEMBLY_CUBE_Mirror_Kinematic_45**					4,61
	ASSEMBLY_CUBE_empty_1×1		1		2,2
	20_Cube_Insert_Kinematic_Mirrormount_45_base	1	1	Link	0,3
	Metal plate 30×40 mm², custom cut	0	1		0
	30×30 mm² Mirror	0	1	Link	0,1
	Screws M3×12	0	3	Link	0,2
	Ball magnets	0	3	Link	0,47
**ASSEMBLY_CUBE_RaspiCam**					33,7
	ASSEMBLY_CUBE_empty_1×1		1		2,2
	20_Cube_Insert_RaspiCam	1	1	Link	0,3
	Raspberry Pi Camera	0	1	Link	27
	Raspberry Pi Camera long cable	0	1	Link	4
	M2 screw + nut	0	2	Link	0,1
**ASSEMBLY_CUBE_S-Stage**					21,75
	10_Lid_1x1_v2_thin	1	1	Link	0,2
	30_Z_Translator_Lightsheet	1	1	Link	0,5
	30_Coupling_Screw_28BYJ_M3	1	1	Link	0
	Screws M3×12	0	6	Link	0,2
	M3×25 screw with nut (non-magnetic)	0	1	Link	0,1
	Metal plate 30×40 mm², custom cut	0	1		0
	30_Syringe_holder	1	1	Link	0
	ESP32	0	1	Link	8
	28-BYJ stepper motor with driver board	0	1	Link	5,35
	Female-Female Jumper Wire	0	6	Link	0,6
	USB-microUSB cable	0	1	Link	2,8
**ASSEMBLY_CUBE_Z-stage**					50,35
	10_Cube_2x1	1	1	Link	0,5
	10_Lid_el_2x1	1	1	Link	0,3
	20_focus_inlet_linearflexure	1	1	Link	0,6
	30_Coupling_Screw_28BYJ_M3	1	1	Link	0
	30_focus_inlet_objective_mount	1	1	Link	0,1
	30_Z_Stage_Adapterplate	1	1	Link	0,3
	30_Z_Stage_Fluomodule	1	1	Link	0,2
	40_XY_Stage_Clamp_Slide	1	1	Link	0,1
	Microscope Objective $m = 10\times,\ NA = 0.3,\ f' \approx 17,6\,mm$	0	1	Link	15,4
	Screws M3×12	0	20	Link	0,6
	Screws M3×8	0	3	Link	0,2
	M3×30 screw with nut (non-magnetic)	0	1	Link	0,1
	Screws M3×18	0	2	Link	0,2
	ESP32	0	1	Link	8
	28-BYJ stepper motor with driver board	0	1	Link	5,35
	Female-Female Jumper Wire	0	6	Link	0,6
	USB-microUSB cable	0	1	Link	2,8
**Raspberry Pi + accesories**					187,8
	Raspberry Pi 4 B	0	1	Link	40
	Raspberry Pi 7" Display	0	1	Link	64,5
	Raspberry Pi Case	0	1	Link	22,3
	Raspi 4 Power supply	0	1	Link	9,5
	SanDisk Ultra microSD card	0	1	Link	11
	Keyboard	0	1	Link	27
	USB hub	0	1	Link	13,5
**ASSEMBLY_CUBE_Eyepiece**				Link	16,11
	ASSEMBLY_CUBE_empty_1×1		1	Link	2,2
	20_Cube_Insert_Holder-okular (Config 2)	1	1	Link	0,2
	Eyepiece 20×	1	1	Link	13,11
**ASSEMBLY_CUBE_Lens**					13,5
	ASSEMBLY_CUBE_empty_1×1		1		2,2
	20_Cube_Insert_Lens_holder	1	1	Link	0,3
	Microscope Objective $m = 4\times,\ NA = 0.14,\ f' \approx 29\,mm$	0	1	Link	11

Table 8. In-incubator microscope: Complete Bill of Materials

Module	Part	Printable	Quantity	Source	Unit price (Euro)
**ASSEMBLY_Baseplate 4×2**					16,34
	Baseplate 4×2	1	1	Link	1,3
	Ball magnets	0	32	Link	0,47
**ASSEMBLY_CUBE_empty_1×1**					2,2
	10_Cube_1x1	1	1	Link	0,4
	10_Lid_1x1	1	1	Link	0,2
	Screws M3×12	0	4	Link	0,2
**ASSEMBLY_CUBE_LED_Matrix**					23,69
	10_Lid_1x1	1	1	Link	0,2
	30_Cube_LED_Array	1	1	Link	0,2
	Screws M3×12	0	4	Link	0,2
	M2 screw + nut	0	2	Link	0,1
	LED Matrix 8×8	0	1	Link	9,99
	ESP32	0	1	Link	8
	Female-Female Jumper Wire	0	3	Link	0,5
	USB-microUSB cable	0	1	Link	2,8
**ASSEMBLY_CUBE_Mirror_45**					2,51
	ASSEMBLY_CUBE_empty_1×1		1	Link	2,21
	20_Cube_Insert_Mirror_Holder_30x30Mirror	1	1	Link	0,2
	30×30 mm² Mirror	0	1	Link	0,1
**ASSEMBLY_CUBE_RaspiCam**					33,71
	ASSEMBLY_CUBE_empty_1×1		1	Link	2,21
	20_Cube_Insert_RaspiCam	1	1	Link	0,3
	Raspberry Pi Camera	0	1	Link	27
	Raspberry Pi Camera long cable	0	1	Link	4
	M2 screw + nut	0	2	Link	0,1
**ASSEMBLY_CUBE_Z-stage**					50,35
	10_Cube_2x1	1	1	Link	0,5
	10_Lid_el_2x1	1	1	Link	0,3
	20_focus_inlet_linearflexure	1	1	Link	0,6
	30_Coupling_Screw_28BYJ_M3	1	1	Link	0
	30_focus_inlet_objective_mount	1	1	Link	0,1
	30_Z_Stage_Adapterplate	1	1	Link	0,3
	30_Z_Stage_Fluomodule	1	1	Link	0,2
	40_XY_Stage_Clamp_Slide	1	1	Link	0,1
	Microscope Objective $m = 10\times,\ NA = 0.3,\ f' \approx 17,6\,mm$	0	1	Link	15,4
	Screws M3×12	0	20	Link	0,6
	Screws M3×8	0	3	Link	0,2
	M3×30 screw with nut (non-magnetic)	0	1	Link	0,1
	Screws M3×18	0	2	Link	0,2
	ESP32	0	1	Link	8
	28-BYJ stepper motor with driver board	0	1	Link	5,35
	Female-Female Jumper Wire	0	6	Link	0,6
	USB-microUSB cable	0	1	Link	2,8
**Raspberry Pi + accesories**					187,8
	Raspberry Pi 4 B		1	Link	40
	Raspberry Pi 7" Display		1	Link	64,5
	Raspberry Pi Case		1	Link	22,3
	Raspi 4 Power supply		1	Link	9,5
	SanDisk Ultra microSD card		1	Link	11
	Keyboard		1	Link	27
	USB hub		1	Link	13,5

9 UC2 Use-cases

The core-idea of the UC2-system is to be open, so that it can be used by a large number of people. In best case, users do not only use the system, but participate actively in the iterative design process by suggesting new applications, finding errors. This can conveniently be done using for example the issue-tracking feature embedded in the GitHub repository. Alternatively private messages, feedback-rounds on workshops or discussions through social media channels such as Twitter can be used for feedback mechanism. After promoting the principle of the UC2 system in a number of talks and workshops, many people started replicating the system. Since we cannot keep track of the number of downloads and actually printed systems, it is hard to track how many people apart from us actually built and used it. Nevertheless, we found the scientific community on Twitter, where we created a dedicated UC2-Twitter account (@openUC2)as a helpful measure and feedback mechanism to track issues, ideas, improvements and to give a rough estimate how many systems are in actual use (exemplary shown in Fig. 23). With the MDK provided through our GitHub repository, we invite people to start developing their own modules and post their own designs by e.g. by forking the repository or sharing it through different channels like Twitter.

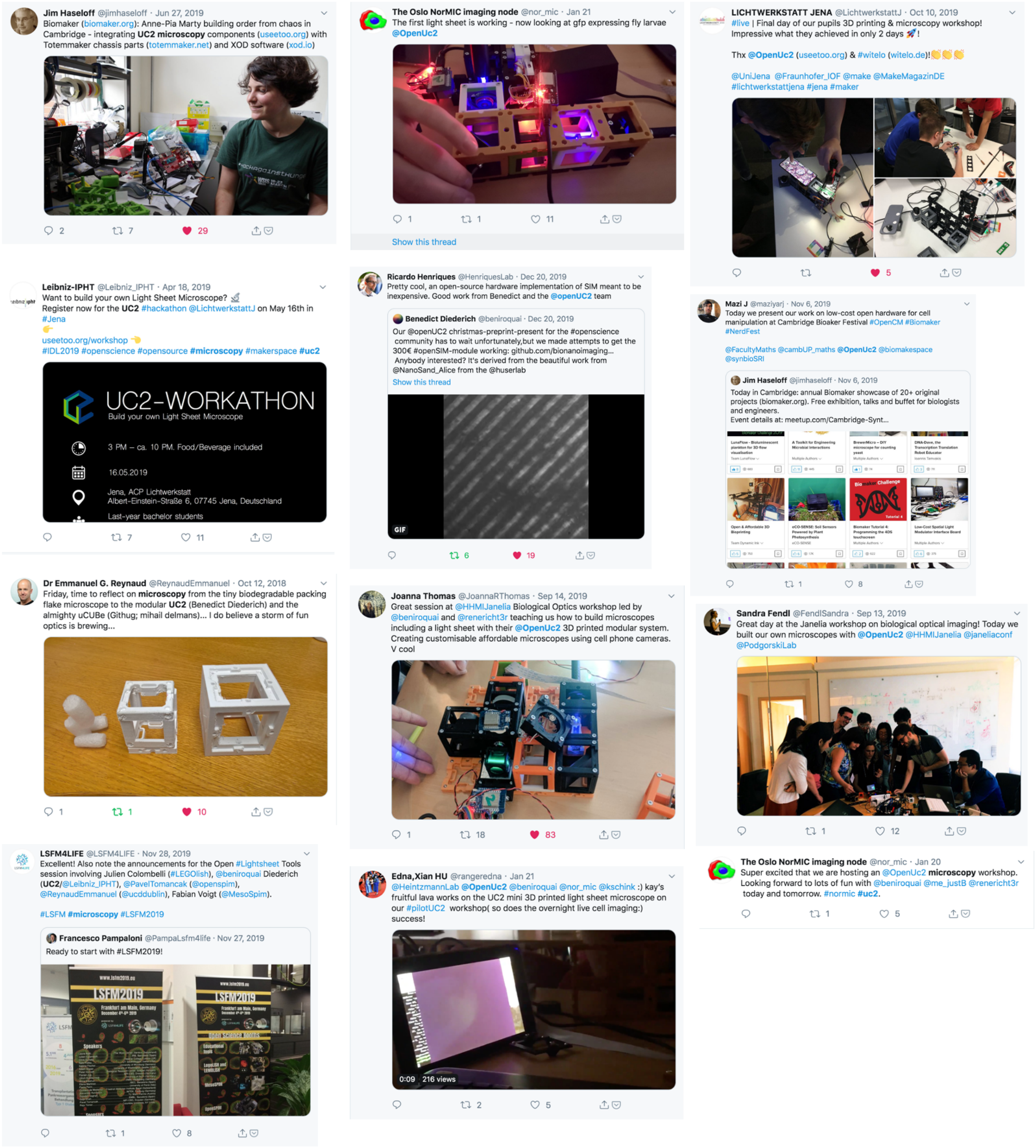

Figure 23. Publicly announced UC2 workshops and use-cases Even though it is hard to track how many UC2 systems are in actual use, we collected an exemplary over-view of some user-feedback and successfully assembled UC2 setups.

From the workshops we found, that it is of great importance, that the entrance threshold is set very low to attract new users to start developing using the UC2 system. In this way, the documentation should be self-explanatory and thus act as a decentralized multiplier.

10 Sample Preparation

10.1 Primary Macrophages

Peripheral blood mononuclear cells (PBMCs) were isolated from healthy volunteer adult donors by Ficoll density centrifugation approved by the ethical committee of the UKJ.

Monocytes were detached and $1.5 \cdot 10^5$ were seeded in a $35\,mm$ rinsed with $3\,ml$ X-Vivo 15 with supplements. The blood was mixed with isobuffer (PBS without Ca/Mg (Gibco), $2\,mM$ EDTA (Sigma Aldrich, St. Louis, USA), 0.1% BSA (Sigma Aldrich) and placed on top of Biocoll (Merck, Darmstadt, Germany) without mixing in a $50\,ml$ tube. Biocoll and Blood were centrifuged with 800 x g for $20\,min$ with out break. The ring of PBMCs was transferred in a new $50\,ml$ tube and washed twice with isobuffer. PBMCs were seeded at a density of $1 \cdot 10^6$ $cells/cm^2$ in X-VIVO 15 medium (Lonza, Cologne, Germany) supplemented with 10% (v/v) autologous human serum, 10 ng/ml granulocyte macrophage colony stimulating factor (GM-CSF) and $10\,g/ml$ macrophage colony stimulating factor (M-CSF) (PeproTech, Hamburg, Germany) and Pen/Strep (Sigma Aldrich). After $1\,h$ PBMCs were washed twice with Roswell Park Memorial Institute (RPMI) and remaining monocytes were then rinsed with X-Vivo with supplements. $16\,h$ after isolation monocytes were washed with prewarmed PBS (w/o Ca/mg) and incubated $7\,min$ with prewarmed with 4 mg/ml lidocain (Sigma Aldrich) and $1\,mM$ EDTA (Sigma Aldrich). Detached monocytes were places in a $15\,ml$ tube and centrifuged $7\,min$ by 350 x g. Sediment monocytes were counted and $1.5 \cdot 10^5$ were seeded in a $35\,mm$ dish and rinsed with $3\,ml$ X-Vivo 15 with supplements. After $24\,h$ were the cells washed once with X-Vivo 15 and the monocytes were rinsed with $3\,ml$ fresh X-Vivo 15 with supplements and placed in the microscope.

10.2 Phagocytosis:

Stimulated cells were washed with PBS. X- with/out supplements containing $0.25\,smg/ml$ pHrodo(TM) Green *E. Coli* BioParticles(TM) Conjugate for Phagocytosis (Thermo Fisher Scientific, MA, USA) was added and imaged for 1h. Fluorescence intensity was analysed.

10.3 HPMEC

HPMEC-eGFP were cultured in Endopan 300SL supplemented with serum substitute Panexin SL-S, EGF, FGF-2, VEGF, Vitamin C, R3-IGF-1, GA, Hydrocortisone, Heparin (Pan Biotech, P04-0065K). Cells were maintained in $6\,cm$ culture dishes in $37\,C°/5\%\ CO_2$ incubator. To prepare the samples, HPMEC-eGFP were passaged at 90 % confluency and 30,000 cells were plated on $12\,mm$ diameter coverslips. After 24 h, cells were fixed in 4 % Paraformaldehyde for $15\,min$ at room temperature and mounted with Mowiol (Sigma Alrdrich, USA) solution.

10.4 Zebrafish larva for light sheet imaging

The zebrafish larva used in the described experiments belongs to transgenic line Tg(kdr.EGFP). It was treated with PTU (Phenylthiourea) in order to be transparent and fixed with PFA (Paraformaldehyde) at the age of 72 hours. The living organism expresses GFP in blood vessels. The fluorescent signal of a fixed tissue is weaker compared to living organism, due to fixation. For the light sheet measurements, $200\,mg$ Agarose (Agarose standard, art. 3810.2, Carl Roth GmbH) was dissolved in $10000\,mg$ H_2O at $160°C$ while stirring. After Agarose was fully dissolved, temperature is reduced to $100°C$. The tip of a syringe (1 ml syringe, Injekt-F from Braun) was cut before we fill the syringe with ca. $0.25\,ml$ of agarose. The sample was placed inside the agarose using a pipette before covering it with a few drops of liquid agarose using. The agarose needs roughly an hour to solidify in the fridge at around $7°C$. The study and experimental protocols used therein were approved by the ethics committee Leibniz Institute on Ageing, Fritz Lipmann Institute (FLI), Beutenbergstraße 11, 07745 Jena, Germany.

10.5 Drosophila larva for light sheet imaging

The drosophila had the w; asl YFP (T2) genotype and it constitutively expressed GFP in alpha-tubulin. It was imaged alive and mounted in the same way as described about for the zebrafish, only with lower concentration of agarose. The study and experimental protocols used

therein were approved by the ethics committee Oslo NorMIC Imaging Platform, Department of Biosciences, University of Oslo, Blindernveien 31, 0371 Oslo, Norway.

10.6 *E. coli* bacteria

A suspension of living *E. Coli* in LB-medium is fixed in 4% PFA in PBS. We add $0.4\mu M$ mCLING Atto647N (Synaptic system, Göttingen, Germany) to the bacteria stock solution and incubated it for 5 minutes. Concentration of the bacteria was achieved using ultra-centrifuging at $10.000\,rounds/min$ for 3 minutes. After aspirating the top layer, 4% PFA is added. Removing unbound dyes is done by repeatedly centrifuging, aspirating the top layer and refilling it with PBS.

Coverslips are prepared by adding 0.01% poly-l-lysine (PLL, Sigma Aldrich) to the surface and incubate it for 30 min at room-temperature. After removing the PLL, the bacteria were added and incubated for another $30\,min$. We washed it $3\times$ using PBS and sealed it on a microscope slide.

Bibliography

1. Hankammer, S., Jiang, R., Kleer, R. & Schymanietz, M. From Phonebloks to Google Project Ara. A Case Study of the Application of Sustainable Mass Customization. *Procedia CIRP* **51,** 72–78 (2016).
2. Diederich, B., Lachmann, R., Uwurukundo, X. & Marsikova, B. *UC2 Github Hardware Repository* `https://github.com/bionanoimaging/UC2-GIT` (2019).
3. Lachmann, R., Diederich, B. & Uwurukundu, X. *UC2 Github Software Repository* `https://github.com/bionanoimaging/UC2-Software-GIT/` (2019).
4. Community. *Kivy - Open source Python library for rapid development of applications that make use of innovative user interfaces, such as multi-touch apps.* `https://kivy.org/%7B%5C#%7Dhome` (2019).
5. Selva, A. *Moquette, V.0.12.1* `https://github.com/moquette-io/moquette` (2019).
6. Elipse. *Eclipse Mosquitto - An open source MQTT broker, V1.16.2* `https://mosquitto.org/` (2019).
7. Booth, M. J., Jesacher, A., Juškaitis, R. & Wilson, T. Full spectrum filterless fluorescence microscopy. *Journal of Microscopy.* doi:`10.1111/j.1365-2818.2009.03317.x` (2010).
8. Sharkey, J. P., Foo, D. C. W., Kabla, A., Baumberg, J. J. & Bowman, R. W. A one-piece 3D printed flexure translation stage for open-source microscopy. *Review of Scientific Instruments* **87.** doi:`10.1063/1.4941068` (2016).
9. Tian, L. & Waller, L. Quantitative differential phase contrast imaging in an LED array microscope. *Optics Express* **23,** 11394 (2015).
10. Royer, L. A. *et al.* Adaptive light-sheet microscopy for long-term, high-resolution imaging in living organisms. *Nature Biotechnology* **34,** 1267–1278 (2016).
11. Banterle, N., Bui, K. H., Lemke, E. A. & Beck, M. Fourier ring correlation as a resolution criterion for super-resolution microscopy. *Journal of Structural Biology* **183,** 363–367 (2013).
12. Schindelin, J. *et al.* Fiji: An open-source platform for biological-image analysis. *Nature Methods.* doi:`10.1038/nmeth.2019` (2012).
13. Lidke, K. A., Rieger, B., Lidke, D. S. & Jovin, T. M. The role of photon statistics in fluorescence anisotropy imaging. *IEEE Transactions on Image Processing.* doi:`10.1109/TIP.2005.852458` (2005).
14. Van Vliet, L. J., Sudar, D. & Young, I. T. Digital Fluorescence Imaging Using Cooled CCD Array Cameras invisible. *Cell Biology* (1998).
15. Li, J. *et al.* High-speed in vitro intensity diffraction tomography. *Advanced Photonics* **1,** 1–13 (2019).
16. Siedentopf, H. & Zsigmondy, R. Uber Sichtbarmachung und Größenbestimmung ultramikoskopischer Teilchen, mit besonderer Anwendung auf Goldrubingläser. *Annalen der Physik* **315,** 1–39 (1902).

17. Pitrone, P. G. *et al.* OpenSPIM: An open-access light-sheet microscopy platform. *Nature Methods* **10,** 598–599 (2013).

18. Reynaud, E. G., Peychl, J., Huisken, J. & Tomancak, P. Guide to light-sheet microscopy for adventurous biologists. *Nature Methods* **12,** 30–34 (Jan. 2014).

19. Müller, C. B. & Enderlein, J. Image Scanning Microscopy. *Physical Review Letters* **104,** 198101 (May 2010).

20. York, A. G. *et al.* Resolution doubling in live, multicellular organisms via multifocal structured illumination microscopy. *Nature Methods* **9,** 749–754 (2012).

21. McGregor, J. E., Mitchell, C. A. & Hartell, N. A. Post-processing strategies in image scanning microscopy. *Methods* **88,** 28–36 (2015).

22. Sheppard, C. J. R., Mehta, S. B. & Heintzmann, R. Superresolution by image scanning microscopy using pixel reassignment. *Optics letters* **38,** 2889–92 (2013).

23. Fuchs, I. *Github: FreedCam* `https://github.com/KillerInk/FreeDcam` (2019).

24. Diederich, B., Wartmann, R., Schadwinkel, H. & Heintzmann, R. Using machine-learning to optimize phase contrast in a low-cost cellphone microscope. *PloS one* **13,** e0192937 (2018).

25. Gross, H., Singer, W., Totzeck, M. & Gross, H. *Handbook of Optical Systems* 1–690. doi:`10.1002/3527606688` (2006).
