# Supplementary material for: A versatile and customizable low-cost 3D-printed open standard for microscopic imaging
Source: Nat Commun. 2020 Nov 25;11:5979. doi: 10.1038/s41467-020-19447-9 (PMC7688980; doi:10.1038/s41467-020-19447-9)
Supplement: Supplementary file 3 — Description of Additional Supplementary Files [file 41467_2020_19447_MOESM3_ESM.docx]

﻿

**Supplementary Video 1.**

Long-term 48h image series with the incubator microscope (10x, 0.32NA objective) at a frame-rate of 1 frame/minute. Incubator-contained measurement of isolated human blood monocytes. The aim was to document differentiation of monocytes to macrophages and analyse their movement pattern without stimulation.

**Supplementary Video 2.**

The video shows long-term imaging of the differentiation of blood-born monocytes to macrophages. Within the time span of seven days the monocytes increase size and are “looking” around. Obvious are the filopodia surround the cells. Moving macrophages become fusiform, elongate and follow their protrusions with the cell body.

**Supplementary Video 3.**

Reconstruction of the complex refractive index of unlabelled cheek cells using the annular intensity diffraction tomography algorithm (aIDT). A number of LEDs on an LED-ring placed at a distance to the sample of ~74mm such that local illumination is approximated as a series of plane waves varying in azimuth. The inverse filtering process can reconstruct a 3D stack of the permittivity distribution. The acquisition was performed using a cellphone camera (Huawei P20, Pro, China) further described in Chapter \ref{sec:sub_experiment_aidt}.

**Supplementary Video 4.**

Through-focus series of a Drosophila larva. Due to the large depth of field of the 4x, NA=0.17 objective, a data stack was acquired by moving the light sheet through a fixed sample i.e. by changing the angle of the kinematic mirror in the illumination path. The GFP-expressing drosophila larva was focussed by the detection path and the illumination plane was then moved through it by changing the tilt of the kinematic mirror. Although the whole three-dimensional sample is in focus, only the illuminated parts yield signal being imaged onto the camera. The video was acquired with a cellphone camera (Huawei P20, Pro, China) and a 20x eyepiece.

Alternatively, one can move the whole sample through the fixed light sheet aligned to the focus-plane using the sample-stage equipped with a flexure bearing. This was done in the video of the GFP-expressing zebrafish larva.

The video was acquired with a Raspberry Pi camera with a lens and a $20\times$ eyepiece.

**Supplementary Video 5.**

The conversion from a simple bright field into a light sheet microscope can be accomplished within less than five minutes using TheBOX. The modules can easily be reused for different imaging modalities. The components are pre-aligned and remain their position when packed again, useful for transporting the whole system.

**Supplementary Video 6.**

Long-term measurements of MDCK-cells at room temperature over night (8h) in an 35mm petri-dish at a UC2 workshop , which took place in Oslo, where participants were able to bring their samples.

**Supplementary Video 7.**

Time-series imaging at ~1 fps of fixed but mobile *E. coli* bacteria using the infinity-corrected fluorescence microscope (see Supp. Section \ref{sec:frc_comparison}). The ATTO647-labelled *E. coli* were illuminated with a coherent entertainment laser ($$\backslash$

$\lambda_{red}=635nm, P_{laser}= 200mW$ move in aqueous solution due to Brownian motion and can nicely be observed with the low-SNR RGB camera from the Raspberry Pi (v2.1). During the ca. 10 minutes experiment, some bacteria start adhering to the cover glass. By increasing the laser intensity inside the UC2 GUI, a dominant bleaching of the bacteria can be observed.
